# Structural Plasticity of GABAergic Pallidothalamic Terminals in MPTP-Treated Parkinsonian Monkeys: A 3D Electron Microscopic Analysis

**DOI:** 10.1523/ENEURO.0241-23.2024

**Published:** 2024-03-19

**Authors:** G. J. Masilamoni, H. Kelly, A. J. Swain, J. F. Pare, R. M. Villalba, Y. Smith

**Affiliations:** ^1^Emory National Primate Research Center, Atlanta, Georgia 30322; ^2^Udall Center of Excellence for Parkinson's Disease, Emory University, Atlanta, Georgia 30322; ^3^Department of Neurology, Emory University, Atlanta, Georgia 30322

**Keywords:** centromedian nucleus, GABAergic, mitochondrial enlargement, pallidothalamic, Parkinson's disease, ventral anterior nucleus

## Abstract

The internal globus pallidus (GPi) is a major source of tonic GABAergic inhibition to the motor thalamus. In parkinsonism, the firing rate of GPi neurons is increased, and their pattern switches from a tonic to a burst mode, two pathophysiological changes associated with increased GABAergic pallidothalamic activity. In this study, we used high-resolution 3D electron microscopy to demonstrate that GPi terminals in the parvocellular ventral anterior nucleus (VApc) and the centromedian nucleus (CM), the two main GPi-recipient motor thalamic nuclei in monkeys, undergo significant morphometric changes in parkinsonian monkeys including (1) increased terminal volume in both nuclei; (2) increased surface area of synapses in both nuclei; (3) increased number of synapses/GPi terminals in the CM, but not VApc; and (4) increased total volume, but not number, of mitochondria/terminals in both nuclei. In contrast to GPi terminals, the ultrastructure of putative GABAergic nonpallidal terminals was not affected. Our results also revealed striking morphological differences in terminal volume, number/area of synapses, and volume/number of mitochondria between GPi terminals in VApc and CM of control monkeys. In conclusion, GABAergic pallidothalamic terminals are endowed with a high level of structural plasticity that may contribute to the development and maintenance of the abnormal increase in pallidal GABAergic outflow to the thalamus in the parkinsonian state. Furthermore, the evidence for ultrastructural differences between GPi terminals in VApc and CM suggests that morphologically distinct pallidothalamic terminals from single pallidal neurons may underlie specific physiological properties of pallidal inputs to VApc and CM in normal and diseased states.

## Significance Statement

Parkinson's disease is associated with complex functional changes in the neuronal communication between the basal ganglia, thalamus, and cerebral cortex. In this study, Masilamoni et al. use cutting-edge 3D high-resolution electron microscopy to demonstrate that axon terminals from the internal globus pallidus to the motor thalamus, one of the main output projections of the basal ganglia, undergo robust structural differences in morphology, size of synapses, and mitochondrial volume in a nonhuman primate model of parkinsonism. These results lay the foundation for a deeper understanding of the structure–function relationships in neuronal connections that may contribute to the development and maintenance of the abnormal increase in pallidal outflow to the thalamus in the parkinsonian state.

## Introduction

The motor signs of Parkinson's disease (PD) are mainly related to the progressive degeneration of the nigrostriatal dopaminergic (DA) pathway. The loss of striatal DA elicits changes in the firing rate and pattern of neurons in the internal globus pallidus (GPi; Filion, 1979; Miller and DeLong, 1988; Hutchison et al., 1994; Wichmann et al., 1999; Galvan et al., 2015), accompanied by an abnormal increase in the GABAergic inhibition of the ventral motor thalamus and resulting in a reduced thalamocortical activity (Albin et al., 1989; DeLong, 1990; Dharmadhikari et al., 2015). There is a compelling evidence that the striatum and the subthalamic nucleus (STN) undergo major morphological and ultrastructural changes accompanied by robust alterations in electrophysiological and plastic properties in rodent and primate PD models of parkinsonism (Ingham et al., 1998; Day et al., 2006; Raju et al., 2008; Mathai and Smith, 2011; Villalba and Smith, 2011; Fan et al., 2012; Mathai et al., 2015; Villalba et al., 2015; Chu et al., 2017). However, despite the evidence for functional and neurochemical changes of basal ganglia outputs to the thalamus in parkinsonism (see above), our understanding of the neuroplastic changes in synaptic microcircuits that could mediate these effects remains unknown. In the present study, we hypothesized that GPi terminals undergo changes in their morphology, number of synapses, and mitochondrial content that may contribute to the maintenance of increased pallidal inhibition upon the ventral motor thalamus and centromedian nucleus (CM) in 1-methyl-4-phenyl-1,2,3,6-tetrahydropyridine (MPTP)-treated parkinsonian monkeys.

Various sets of observations lay the foundation for this hypothesis as follows: (1) GPi terminals display ultrastructural features (large volume, large number of mitochondria, and multisynaptic innervation) tailored to maintain synaptic inhibition even at high presynaptic firing rates (Bodor et al., 2008; Wanaverbecq et al., 2008). (2) Recent findings from the STN showed that increased pallidal GABAergic inhibition upon STN neurons is associated with an increased number of synapses formed by individual GABAergic terminals from the external globus pallidus (GPe) in the 6-OHDA-treated mouse model of parkinsonism (Fan et al., 2012; Chu et al., 2017). (3) The GABA release from GPi-like terminals in the thalamus and other brain regions displays a low incidence of synaptic depression even when stimulated at abnormally high firing rate (Telgkamp et al., 2004; Wanavebecq et al., 2008; Rudolph et al., 2015). (4) The amount of neurotransmitter release and the number of synapses formed by multisynaptic GABAergic boutons are tightly correlated (Rudolph et al., 2015). (5) Mitochondria are the essential regulators of synaptic transmission and firing rate homeostasis since they are a major source of energy (ATP and NAD+) required for the maintenance and restoration of ion gradients (Duchen, 2000; Nicholls and Budd, 2000; Toescu, 2000; Ruggiero et al., 2021). These observations give a detailed ultrastructural analysis of morphological changes in GPi terminals undergoing the state of parkinsonism will provide a solid substrate for future studies of structure–function relationships of the pallidothalamic system in normal and diseased states.

To address this issue, we used the serial block face scanning electron microscopy (SBF-SEM) approach (Ventura and Harris, 1999; Denk and Horstmann, 2004) and the Reconstruct software (NIH) to build 3D-reconstructed images and quantitatively analyze the morphometry of GABAergic pallidal terminals, synapses, and mitochondrial morphology in the GPi-receiving regions of the motor thalamus in control and parkinsonian monkeys.

The results of these studies have been presented in abstract forms (Masilamoni et al., 2021).

## Materials and Methods

### Animals

Four adult male rhesus monkeys (*Macaca mulatta*, 4.5–8.5 kg) from the Emory National Primate Research Center colony were used in this study (Table 1). All procedures were approved by Emory's Animal Care and Use Committee in accordance with guidelines from the National Institutes of Health. The animals were housed in a temperature-controlled room and exposed to a 12 h light/dark cycle. They were fed twice daily with monkey chow supplemented with fruits or vegetables. The animals had *ad libitum* access to water.

**Table 1.** Subject demographics and clinical data

Monkey	Age (years)	Gender	MPTP dosage (mg/kg)	Cumulative MPTP (mg)	Parkinsonism rating scale	Clinical status
A	3	M	NA	NA	0/30	Naive
B	4	M	NA	NA	0/30	Naive
C	4	M	0.3–0.7	18.15	11/30	Moderate parkinsonism
D	5	M	0.4–0.8	8.2	16/30	Moderate parkinsonism

Monkeys A and B, controls. Monkeys C and D, MPTP-treated. Intramuscular MPTP injection once a week. Parkinsonism rating scale ranges from 0 to 30: 0–4, no impairment; 5–10, mild impairment; 11–20, moderate impairment; 21–30, severe impairment. Data are mean of 3 or more behavioral assessments to determine stability of the model. M, male.

### MPTP administration and evaluation of parkinsonism

The four rhesus monkeys used in this study were divided into two groups: two monkeys were drug-naive and healthy and served as experimental controls. The other two monkeys were rendered moderately parkinsonian via a chronic treatment with MPTP. The two monkeys in the MPTP treatment group received repeated injections (intramuscular) of low doses of MPTP (0.3–0.8 mg/kg; Sigma-Aldrich) delivered at least 1 week apart until a moderate and stable state of parkinsonism emerged. The cumulative drug doses and total duration of the MPTP treatment for each subject are described in Table 1. The methods for evaluation of parkinsonism were described in our previous studies (Masilamoni et al., 2010, 2011, 2022; Mathai et al., 2015). In brief, the animals were habituated to a behavior cage equipped with infrared beams, and their spontaneous movements within this cage were video recorded and monitored for 15 min weekly during the MPTP treatment period. Their movements were scored by two expert observers (one of them blinded to the MPTP treatment regimen) according to a nine-criteria parkinsonism rating scale, with evaluations of the gross motor activity, balance, posture, arm bradykinesia, arm hypokinesia, leg bradykinesia, arm hypokinesia, arm tremor, and leg tremor. Each criterion received a score of 0–3 (normal/absent to severe), for a maximal score of 30. Additionally, infrared beam breaks were counted and compared with baseline numbers measured during the pre-MPTP phase in the same subject. The animals were considered stably parkinsonian once they had achieved a score of 10 or higher on the rating scale and a >60% reduction in beam breaks from the baseline, both persisting over 6 weeks following cessation of the MPTP treatment. The differences in the rating scores between the two observers were <6%. The mean values obtained by the two experimenters were used for the study. Using this approach, the final rating scores for the two MPTP-treated subjects in this study ranged from 10 to 14, corresponding to moderate parkinsonism.

### Anterograde labeling of pallidothalamic terminals

In the two control and two MPTP-treated monkeys, pallidothalamic terminals were labeled anterogradely with viral vector injections in the GPi (Swain et al., 2020). Given the evidence that AAV5 vectors expressing various fluorescence reporter genes are widely used for anterograde circuit mapping of highly interconnected nuclei such as the thalamus and putamen (Samaranch et al., 2017; Evers et al., 2018; Lanciego and Wouterlood, 2020), a total of 2–8 µl of AAV5-hSyn-ChR2-EYFP or AAV5-hSyn-Arch3-EYFP was delivered in the GPi. In the control monkeys, the injections were made under the isoflurane anesthesia with the animal fixed in a stereotaxic frame using aseptic surgical procedures. Preoperative MRI scans of these monkeys were performed to help define the stereotaxic coordinates. Small holes were drilled in the skull, and a Hamilton microsyringe was used to inject the viral vector at a single site in the ventrolateral part of GPi. To deliver the viral vector solution, the plunger of the syringe was pressed manually at an approximate rate of 1 µl/5 min. After completion, the syringe was left in place for 10 min before withdrawing. At the end of the surgery, the skin was sutured, and the animals were treated with analgesics. The animals were allowed to survive for at least 6 months after injection.

To optimize the use of nonhuman primates, the two MPTP-treated parkinsonian monkeys used in the present study were animals that underwent in vivo electrophysiology recordings of the GPe and GPi neuronal activity before receiving AAV5 injections in the GPi. These two monkeys did not undergo any other procedure or received any drugs besides the collection of the single-unit recording data in the normal and parkinsonian state. In these animals, the viral vector solutions were delivered in the GPi using extracellular recordings as a guide to delineate the borders of the neighboring nuclei using procedures previously described from our laboratory (Kliem et al., 2007; Galvan et al., 2010). Preparatory to the injections, recording chambers were stereotactically directed at the pallidum on either side of the brain, placed at an angle of 40° from the vertical in the coronal plane. The chambers were then affixed to the skull with dental acrylic, along with metal holders for head stabilization. Metal screws were used to anchor the acrylic to the bone. During sessions conducted 2–3 weeks’ postsurgery, electrophysiological mapping served to outline the borders of GPe and GPi. GPi cells were identified based on the depth of the electrode (at least 2 mm ventral to the first GPe unit), the presence of “border” cells between GPe and GPi (DeLong, 1971), and the presence of neurons that fired at high frequency, characteristic for GPi cells (DeLong, 1971; Galvan et al., 2005, 2011). The subsequent injections were done using a probe in which the injection tubing was combined with a recording microelectrode (Kliem and Wichmann, 2004). Extracellular recordings were conducted while lowering the injection system to help define the final location of the injections in the GPi (Kliem et al., 2007; Galvan et al., 2010). A microsyringe connected to a pump was used to deliver the viral vector solutions at a rate of 0.1–0.2 μl/min. At the end of the injection, the injectrode was left in place for 10 min before withdrawing. These animals were allowed to survive for 1.5–7 months after the viral vector injections.

### Tissue collection and processing for microscopy

At the completion of the study, the animals were killed with an overdose of pentobarbital and transcardially perfused with a Ringer's solution and a mixture of paraformaldehyde (4%) and glutaraldehyde (0.1%). The brains were removed from the skull, postfixed in 4% paraformaldehyde, and cut in serial sections (60 µm) with a vibratome. The sections were stored at −20°C until further histological processing. Thalamic tissue sections containing the GPi-receiving parvocellular ventral anterior (VApc) or the CM nuclei were removed from the antifreeze solution and placed in phosphate-buffered saline (PBS, 0.01 M), pH 7.4. Adjacent sections immunostained for calbindin D28k (Swain et al., 2020) and the rhesus monkey stereotaxic brain atlas (Paxinos et al., 1999) were used to help delineate the location of these nuclei.

**Figure 1.**
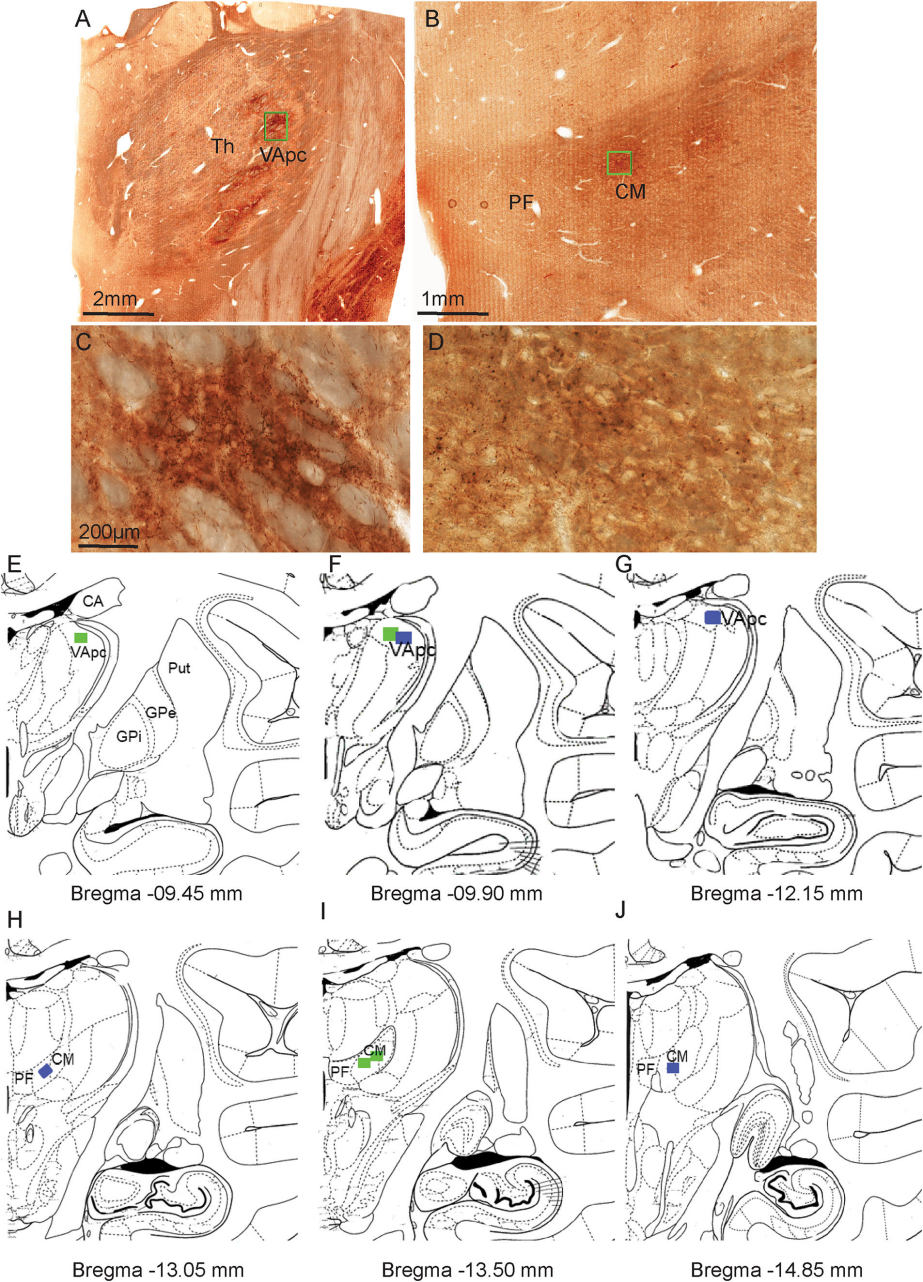
Representative light microscopic images of osmium-fixed sections to show expression of EYFP-labeled terminals in the VApc (***A***) and CM (***B***) after AAV5 injections in GPi. The boxed area in ***A*** and ***B*** are shown at higher magnification in ***C*** and ***D***. ***E–J***, Schematics of coronal sections from the rhesus monkey brain atlas (Paxinos et al., 1999) to show the location of tissue samples taken for SBF/SEM processing (green and blue rectangles indicate control and MPTP monkey, respectively). The AP levels of the different sections are indicated below each panel. CA, caudate nucleus; Put, putamen; Gpe, external globus pallidus; GPi, internal globus pallidus; VApc, parvocellular ventral anterior nucleus; CM, centromedian nucleus.

Selected sections were prepared for the electron microscopy as follows: they were placed in a cryoprotectant solution [phosphate buffer (PB), 0.01 M, pH 7.4, with 25% sucrose and 10% glycerol] for 20 min, frozen at −80°C for 20 min, thawed, and returned to a graded series of cryoprotectant solution (100, 70, 50, 30%) diluted in PBS. The sections were washed in PBS and then preincubated in a solution of 1% normal goat serum and 1% bovine serum albumin in PBS for 1 h. The sections were then immunostained with a green fluorescent protein (GFP) antibody that also recognizes EYFP (Table 2; AB_221569) to localize anterogradely EYFP-labeled GPi terminals in the VApc and CM. This tissue was incubated with a primary rabbit GFP antibody (1:5,000 dilution) for 48 h at 4°C. Next, the sections were rinsed in PBS and transferred for 1.5 h to a solution with a secondary biotinylated goat anti-rabbit antibody (1:200 dilution). After, the sections were placed in a solution of 1% avidin–biotin–peroxidase complex (Vector Laboratories), washed in PBS and Tris buffer, pH 7.6 (0.05 M), and transferred to a solution containing 0.01 M imidazole, 0.005% hydrogen peroxide, and 0.025% 3,3′-diaminobenzidine (DAB) tetrahydrochloride (Sigma-Aldrich) in Tris buffer for 10 min. Several rinses of the tissue in PBS ended the DAB reaction. The sections with the maximum amount of GFP immunostaining in VApc and CM (Fig. 1) were put into vials in phosphate buffer solution and sent to Lerner Research Institute's 3DEM Core at the Cleveland Clinic (Cleveland, Ohio) in 4% paraformaldehyde for SBF-SEM processing. In preparation for the SBF-SEM, the tissue went through a multiday staining process beginning with washing off aldehydes in sodium cacodylate buffer (0.1 M). The tissue samples were then incubated in 1.5% potassium ferrocyanide and 2% osmium tetroxide (in sodium cacodylate buffer, 0.1 M) at 4°C, rinsed in double-distilled H_2_O (ddH_2_O), and incubated in 1% thiocarbohydrazide at 60°C, followed by washes in ddH_2_O. Next, the tissue was placed in 2% osmium tetroxide on a rotator at room temperature, rinsed in ddH_2_O, and left at 4°C for 48–72 h in a saturated aqueous solution of uranyl acetate and then lead stained at 60°C, rinsed in ddH_2_O, and dehydrated in a series of graded ethanol and propylene oxide. Finally, the tissue was placed in the resin (EMbed-812), first a mixture of resin and propylene oxide (50/50) and after that in fresh resin (100%) and embedded in Pelco silicone mold at 60°C until the resin was fully cured (8–14 h). Next, the resin blocks were removed from the mold, trimmed, and mounted on an aluminum pin where the vertical sides of the sample were covered with colloidal silver liquid. Once the imaging surface of the tissue was exposed, the sample was then set up in the SBF-SEM in-chamber ultramicrotome and examined in the electron microscope. Low-, medium-, and high-resolution 2D images were initially taken of the block face surface to assess the tissue preservation. Multiple series of 200–300 EM images were then obtained using two SBF-SEM systems: a Zeiss Sigma VP scanning EM with Gatan 3View system and a Thermo Fisher Scientific Teneo VolumeScope.

**Table 2.** Primary antibodies used in this study

Antigen	Immunizing species	Vendor (primary)	Antibodies dilution used	Secondary antibodies dilution used	Vendor (secondary)	Purpose	RRID
Tyrosine hydroxylase (TH)	Mouse	Millipore, catalog #MAB318	1:1,000	Horse anti-mouse 1:200 (biotinylated)	Vector, catalog #BA2000	To identify dopamine- containing neurons and terminals	AB_2201528
Calbindin D28k (Cb)	Goat	R&D Systems, catalog #AF3320	1:1,000	Horse anti-goat 1:200 (biotinylated)	Vector, catalog #BA9500	To delineate thalamic nuclei	AB_2254256
GFP	Rabbit	Life Technologies, catalog #A11122	1:5,000	Goat anti-rabbit 1:200 (biotinylated)	Vector, catalog #BA9400	To identify EYFP-positive elements (EYFP is a variant of GFP)	AB_221569

### 3D reconstruction from serial sectioning electron microscopy

**Figure 2.**
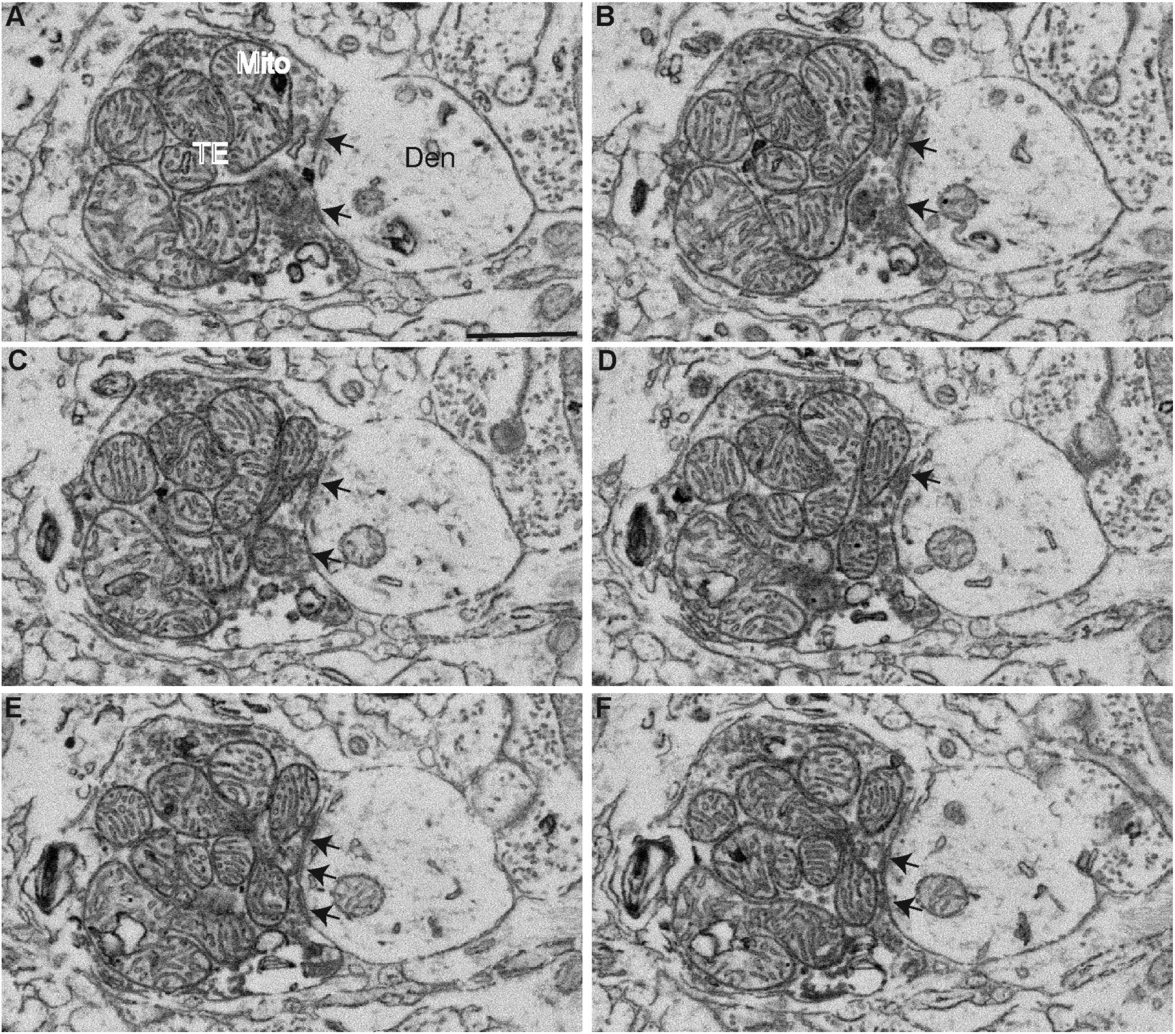
Serial FIB/SEM images of symmetric synapses (arrows) formed by a GPi-like terminal in the VApc of a control monkey. Synapse classification was based on the examination of the full sequence of serial images. Scale bars: ***A–H***, 1 μm.

Selected areas in the VApc and CM with dense GFP-labeled GPi terminals (Fig. 1*A–D*) were used to obtain serial ultrastructural images using an SBF/SEM approach. Approximately 200–400 serially scanned micrographic images (∼70 nm thick) were collected from each region of interest, and labeled and unlabeled GPi terminals were chosen at random from these images to be reconstructed using the 3D software Reconstruct (NIH and synapses.clm.utexas.edu). To avoid bias in the selection of elements being reconstructed, two experimenters, one of them blinded to the treatment (control vs MPTP-treated), reconstructed and analyzed individual terminals. The identification of axon terminal subtypes in the VApc and CM was based on ultrastructural features previously reported in EM studies of the mammalian motor thalamus (Ilinsky et al., 1997; Kultas-Ilinsky et al., 1997; Jones, 2002; Bodor et al., 2008) and in EM studies from our laboratory (Swain et al., 2020). Small (i.e., ∼0.5–0.7 μm in diameter) terminals forming asymmetric synapses were categorized as originating from the cerebral cortex; small- to medium-sized (∼0.5–1.5 μm in diameter) terminals forming single symmetric synapses were considered as putative nonpallidal GABAergic boutons; and large (∼1–3 μm in diameter) terminals enriched in mitochondria (∼1–3 μm in diameter) forming multiple symmetric synapses with single postsynaptic targets were categorized as putative GABAergic terminals from the GPi. In addition, the presynaptic vesicle-filled dendrites of GABAergic interneurons (also referred to as F2 terminals in previous studies; Montero, 1986; Wang et al., 2002) that frequently form symmetric synapses and receive excitatory and inhibitory synaptic inputs were easily distinguishable from other axon terminals. For each terminal category, “synapses” were defined as circumscribed membrane specializations with dense material in a wide synaptic cleft between the presynaptic and postsynaptic membranes with aggregates of vesicles at the presynaptic membrane (Fig. 2). These synaptic membrane specializations were distinct from the nonsynaptic puncta adherentia, another type of appositions between GPi terminals and thalamic cells (Ilinsky et al., 1997; Swain et al., 2020), characterized by symmetric deposits of electron-dense material in both elements and thin space between the two plasma membranes that lack presynaptic vesicle accumulation on either side of the junction (Bodor et al., 2008). Synaptic specializations, which appeared noncontinuous after 3D reconstructions, were treated as separate synapses (Fig. 2).

**Figure 3.**
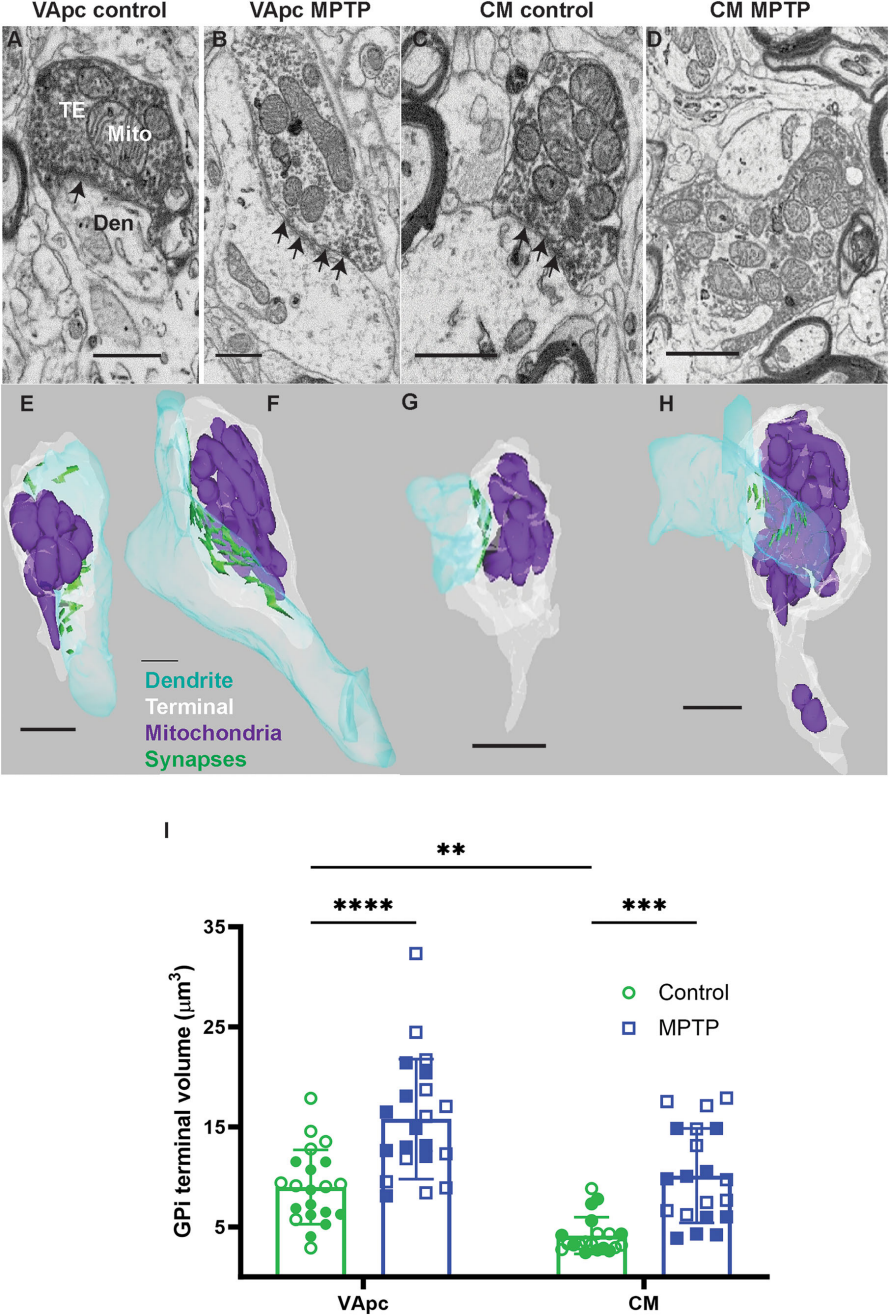
Electron micrographs (***A–D***) and corresponding 3D EM reconstructions (***E–H***) of GFP-positive (***A***,***C***) or negative (***B***,***D***) GPi terminals (TE) that form multiple symmetric axodendritic synapses (***A***, arrow) in the VApc (***A***,***B***) and CM (***C***,***D***) of control or MPTP-treated parkinsonian monkeys. Den, dendrite; Mito, mitochondria. Scale bars: ***A–H***, 1 μm. ***I***, Scatter dot plot with bar graphs comparing the relative volume of GPi terminals in the VApc and CM between control and MPTP-treated parkinsonian monkeys. Each data point is a terminal (VApc, *n* = 21, and CM, *n* = 20). The filled and empty symbols represent data points from the two animals in each group. Statistical differences were determined by two-way ANOVA for repeated measures followed by the Sidak post hoc test. Significance was taken at *p* < 0.05*, *p* < 0.001**, and *p* < 0.0001***. All results are expressed as mean ± standard deviation (SD).

These ultrastructural features, combined with the morphometric data collected from the EYFP-labeled pallidothalamic terminals (Fig. 3*A–D*), further confirmed that GPi is the source of both labeled and unlabeled terminals forming multiple symmetric synapses in VApc and CM of the control and parkinsonian monkeys. TIFF images of serial sections from at least 20 terminals/animal were imported into Reconstruct and calibrated with the section thickness and pixel size provided by the Cleveland Clinic SBF-SEM service core of the Lerner Research Institute. Finally, labeled and unlabeled terminals, mitochondria, synapses, and dendrites from each object analyzed were manually traced in each serial electron micrograph using Reconstruct (Villalba and Smith, 2010, 2011). From these serially identified elements, the software created a 3D representation of each object from which it calculated the volume of terminals, mitochondria, and the surface area (SA) of synapses. Only terminals that could be seen through their full extent in serial sections were reconstructed; a series of 30–100 scanned ultrathin images were used depending on the size of terminals.

Further, we used the mitochondrial complexity index (MCI) to quantify changes in the mitochondrial shape complexity (Vincent et al., 2019; Faitg et al., 2021). The MCI was calculated using the following formula MCI = SA^3^/16π^2^*V*^2^ where SA is the surface area and *V* is the volume of the mitochondria. This equation is used to assess the mitochondrial morphological complexity irrespective of volume (Vincent et al., 2019), thereby providing a quantitative parameter to characterize dissimilarities in mitochondrial morphology between control and parkinsonian states.

### Statistical analysis

The data were statistically analyzed using the GraphPad Prism software (version 9.3). Because of the limited number of monkeys (two control, two MPTP treated) used in this study, individual data points about the terminal volume, the number and volume of synapses made by single terminals, and the number and volume of the mitochondria within a terminal were used for statistical analyses. To reduce the likelihood that the data from one animal drove statistical group differences, unpaired *t* tests were achieved to determine variability between mean values and variances from animals in the same group. This analysis revealed no significant differences between the data from the two control monkeys for both VApc and CM and for the VApc data in the two MPTP-treated animals (Table 3). However, a significant difference was found between values collected from CM in the two MPTP-treated monkeys (Table 3). To provide a detailed account of data collected from each monkey, different symbols were used to illustrate individual data points for each control (green circle and filled circle) and MPTP-treated monkey (blue square and filled square). Multiple comparisons for two-way ANOVA for repeated measures followed by Sidak's post hoc test was used to compare terminal volume, SA of synapses, mitochondria volume, and number of synapses or mitochondria per terminal, between control and MPTP treatments. Significance was taken at *p* < 0.05*, *p* < 0.001**, and *p* < 0.0001***. All results are expressed as mean ± standard deviation (SD). Correlations between the number and size of synapses or mitochondria and terminal volumes were calculated using the linear regression in both control MPTP-treated monkeys.

**Table 3.** Statistical table

Figure	Graph	Data structure	Type of test	Multiple comparison	*p* Values	Power (95% CI of diff)
Figure 1	1*I*	Scatter dot plot with bar	Unpaired *t* test: control VApc, CM	VApc:control 1 vs control 2	0.1527	−5.656 to 0.9567
				CM:control 1 vs control 2	0.7063	−1.448 to 2.093
Figure 1	1*I*	Scatter dot plot with bar	Unpaired *t* test: MPTP VApc, CM	VApc:MPTP 1 vs MPTP 2	0.4187	−7.847 to 3.412
				CM:MPTP 1 vs MPTP 2	0.0112*	−8.895 to −1.306
Figure 1	1*I*	Scatter dot plot with bar	Two-way ANOVA; Sidak post hoc	VApc:control vs VApc:MPTP	<0.0001***	−10.58 to −3.254
				VApc:control vs CM:control	0.0038**	1.444 to 8.770
				CM:control vs CM:MPTP	0.0003***	−9.654 to −2.329
Figure 2	2*A*	Scatter dot plot with bar	Two-way ANOVA; Sidak post hoc	VApc:control vs VApc:MPTP	>0.9999	−6.558 to 5.458
				VApc:control vs CM:control	0.0223*	0.6423 to 12.66
				CM:control vs CM:MPTP	0.0005***	−15.26 to −3.242
	2*B*	Scatter dot plot with bar	Two-way ANOVA; Sidak post hoc	VApc:control vs VApc:MPTP	0.0880	−0.08041 to 0.003388
				VApc:control vs CM:control	0.0028**	−0.09856 to −0.01476
				CM:control vs CM:MPTP	<0.0001***	−0.1370 to −0.05322
Figure 3	3*A*	Scatter dot plot with bar	Two-way ANOVA; Sidak post hoc	VApc:control vs VApc:MPTP	<0.0001***	−2.672 to −0.7560
				VApc:control vs CM:control	0.0039**	0.3065 to 2.247
				CM:control vs CM:MPTP	0.0003***	−2.550 to −0.5883
	3*B*	Scatter dot plot with bar	Two-way ANOVA; Sidak post hoc	VApc:control vs VApc:MPTP	0.9438	−4.705 to 2.389
				VApc:control vs CM:control	0.2390	−0.8735 to 6.310
				CM:control vs CM:MPTP	0.1240	−6.706 to 0.4774
Figure 4	4*A*	Scatter dot plot with bar	Two-way ANOVA; Sidak post hoc	VApc:control vs VApc:MPTP	0.0101*	−1.013 to −0.09417
				VApc:control vs CM:control	0.5965	−0.7124 to 0.2059
				CM:control vs CM:MPTP	0.4541	−0.7457 to 0.1726
	4*B*	Scatter dot plot with bar	Two-way ANOVA; Sidak post hoc	VApc:control vs VApc:MPTP	0.8870	−7.460 to 3.330
				VApc:control vs CM:control	0.4771	−2.117 to 8.803
				CM:control vs CM:MPTP	0.0123*	−11.76 to −0.9751
Figure 5	5*I*	Scatter dot plot with bar	Two-way ANOVA; Sidak post hoc	VApc:control vs VApc:MPTP	0.3823	−0.1730 to 0.8545
				VApc:control vs CM:control	0.3176	−0.8744 to 0.1531
				CM:control vs CM:MPTP	0.5932	−0.2295 to 0.7980
	5*J*	Scatter dot plot with bar	Two-way ANOVA; Sidak post hoc	VApc:control vs VApc:MPTP	0.1294	−0.007909 to 0.1052
				VApc:control vs CM:control	0.9881	−0.07002 to 0.04309
				CM:control vs CM:MPTP	>0.9999	−0.05307 to 0.06004

*p < 0.05, **p < 0.005, ***p <0.005.

## Results

### Nigrostriatal dopamine denervation in MPTP-treated monkeys

Chronic low-dose MPTP exposure was used to slowly induce progressive parkinsonian motor signs and nigrostriatal dopaminergic denervation in two monkeys used in the present study. A detailed description of the MPTP treatment protocol, quantitative data about parkinsonian motor scores, extent of striatal dopamine denervation, and nigral dopaminergic neuron loss are provided in our previous study (Swain et al., 2020). In brief, the postcommissural and lateral edge of the precommissural putamen exhibited the most severe reduction in tyrosine hydroxylase (TH) immunoreactivity, followed by the head and body of the caudate nucleus, which were also significantly affected, while immunoreactivity was much less reduced in the nucleus accumbens. At the midbrain level, the ventral tier of the substantia nigra pars compacta (SNc) were severely damaged, whereas a significant number of TH-immunoreactive neurons and processes remained in the ventral tegmental area and dorsal tier SNc (Swain et al., 2020). These results are consistent with previous findings from our laboratory using the same animal model (Masilamoni et al., 2010, 2011).

### Pallidothalamic terminals in VApc and CM undergo robust ultrastructural changes in MPTP-treated parkinsonian monkeys

Anterogradely labeled pallidothalamic terminals in VApc and CM of control and MPTP-treated monkeys were identified by the expression of EYFP immunoreactivity. As expected based on previous studies (Ilinsky et al., 1997; Kultas-Ilinsky et al., 1997; Sidibe et al., 1997), these terminals were large (1.0–3.0 μm in diameter), enriched in mitochondria, and formed multiple synapses predominantly with dendritic profiles devoid of synaptic vesicles (i.e., of projection neurons) in VApc (Fig. 3*A*,*B*) and CM (Fig. 3*C*,*D*). From this material, we randomly selected and reconstructed a minimum of 40 labeled GPi terminals (at least 10/animal) out of the two control and the two parkinsonian animals, respectively, with an *x*–*y* resolution of 7 nm and a *z* resolution of 70 nm (30–100 serial images per GPi terminal). A minimum of 40 additional unlabeled GPi-like terminals (at least 10 from each nucleus in control and MPTP-treated condition) were reconstructed and added to the sample size. Each pallidothalamic terminal was manually traced in its full extent through a series of images which were then uploaded in the Reconstruct software to build 3D structures (Fig. 3*E–H*). From these reconstructed terminals, we measured and compared their volume between VApc and CM in control and parkinsonian monkeys. This analysis revealed a significant increase in the average volume of individual GPi terminals in the VApc and CM of MPTP-treated monkeys relative to controls (Fig. 3*I*; Table 3). Furthermore, our 3D quantitative analysis demonstrated that the GPi terminal volume in the control VApc (mean 8.99 µm^3^) was significantly larger than that in CM (Table 3; mean 4.14 µm^3^).

### Morphometric changes of pallidothalamic synapses in parkinsonian monkeys

**Figure 4.**
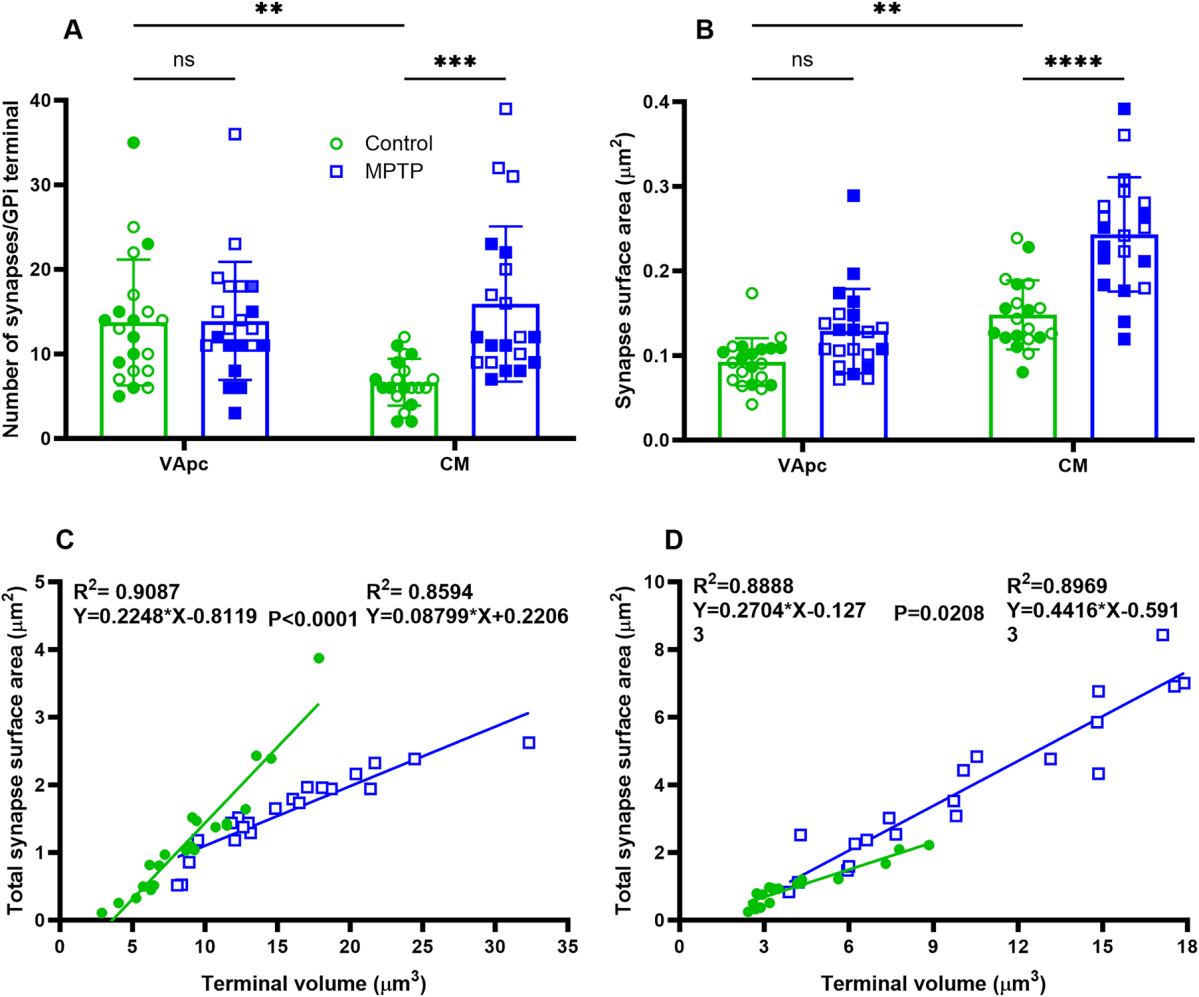
***A***, ***B***, Comparison of synapses number and SA of GPi terminals in VApc and CM between control and parkinsonian monkeys. No significant difference was found in the number of synapses and SA of GPi terminals in VApc between control and parkinsonian monkeys. In CM, the number of synapses and SA per GPi axon terminals is significantly larger in parkinsonian monkeys than that in controls (one-way ANOVA with Sidak's post hoc test; ***p = 0.0005; ****p < 0.0001). GPi terminals in VApc have a greater number of synapses than those in CM (*p* = 0.02; ***A***), but the synapses of GPi terminals in CM have a larger SA than those in VApc (*p* < 0.002; ***B***). ***C***, ***D***, Scatter diagrams showing the linear regression analysis of terminal volume (μm^3^) and total synapses SA (μm^2^) for control (green) and parkinsonian monkeys (blue) in VApc (***C***; *n* = 21) and CM (***D***; *n* = 20). In all cases, the terminal volume was positively correlated with the total synapses SA. Significant differences in slopes were found between the control and parkinsonian monkeys in both VApc and CM (*p* < 0.0001; *p* = 0.0208; ***C***,***D***).

The number, size, and shape of synapses are key structural determinants of the synaptic efficacy, strength, and short-term and long-term plasticity (Matz et al., 2010; Sudhof, 2012; Wilhelm et al., 2014; Holler et al., 2021). To determine if the morphometry of pallidothalamic synapses was altered in parkinsonian monkeys, we used the 3D SBF-SEM reconstruction approach to quantify the number and SA of symmetric synapses formed by individual GPi terminals in control and MPTP-treated parkinsonian monkeys. From the 80 reconstructed pallidal terminals described above (Fig. 3), a total of 399 (266/133 in control VApc/CM) and 595 (277/318 in parkinsonian VApc/CM) synapses were morphometrically analyzed. The total number and SA of synapses formed by individual GPi terminals were significantly increased in CM of parkinsonian monkeys (Fig. 4*A*,*B*; Table 3). However, there was no significant difference in the number and SA of synapses in the VApc between normal and MPTP-treated monkeys (Fig. 4*A*,*B*). In controls, the median number of synapses established by single terminals was 13.3 in VApc (*n* = 21; minimum to maximum, 5–35) and 6.65 in CM (*n* = 20; minimum to maximum, 3–12; Fig. 4*A*,*B*). In each case, all synapses formed by individual terminals converged on a single postsynaptic target.

Our 3D EM analysis also revealed a significant difference in the number and SA of synapses between the VApc and CM of control monkeys such that GPi terminals in the VApc harbored a larger number of synapses than those in CM (Table 3), but pallidothalamic synapses in CM have a larger SA than those in VApc (Fig. 4*A*,*B*; Table 3). These structural differences were not found in parkinsonian monkeys. To determine if the morphology and prevalence of synapses were related to the size of GPi terminals, we made correlations between the total number or SA of synapses and the volume of GPi terminals. Positive correlations were found between the GPi terminal volume and the SA of synapses in both VApc and CM of control and parkinsonian monkeys (Fig. 4*C*,*D*). However, the regression lines for the control and MPTP-treated group have significantly different intercepts and slopes in VApc (Fig. 4*C*) and less so in CM (Fig. 4*D*).

### Mitochondrial morphology is significantly altered in pallidothalamic terminals of parkinsonian monkeys

Mitochondria in axon terminals are critical for the mobilization of the reserve pool of synaptic vesicles and for the regulation of synaptic strength (Shepherd and Harris, 1998; Rowland et al., 2000; Billups and Forsythe, 2002; Verstreken et al., 2005; Gazit et al., 2016; Smith et al., 2016; Cserep et al., 2018). Alterations in the mitochondrial size, shape, and number are frequently encountered in neurological diseases (Whiting et al., 1979; Mortiboys et al., 2008; Guo et al., 2017; Trinh et al., 2021). GPi terminals are enriched in the mitochondria (Fig. 3). Because of the high and tonic firing rate of GPi neurons, the neurotransmitter release and neuroplastic properties of pallidothalamic terminals are highly dependent on a constant and reliable supply of energy through the mitochondrial respiration. Given the recent evidence that changes in mitochondrial morphology and prevalence may contribute to the pathophysiology of brain disorders (Trimmer et al., 2000; Brustovetsky et al., 2021; Liu et al., 2021; Toomey et al., 2022), we used the 3D SBF/SEM approach to compare mitochondrial morphology in pallidothalamic terminals in VApc and CM between control and parkinsonian monkeys.

**Figure 5.**
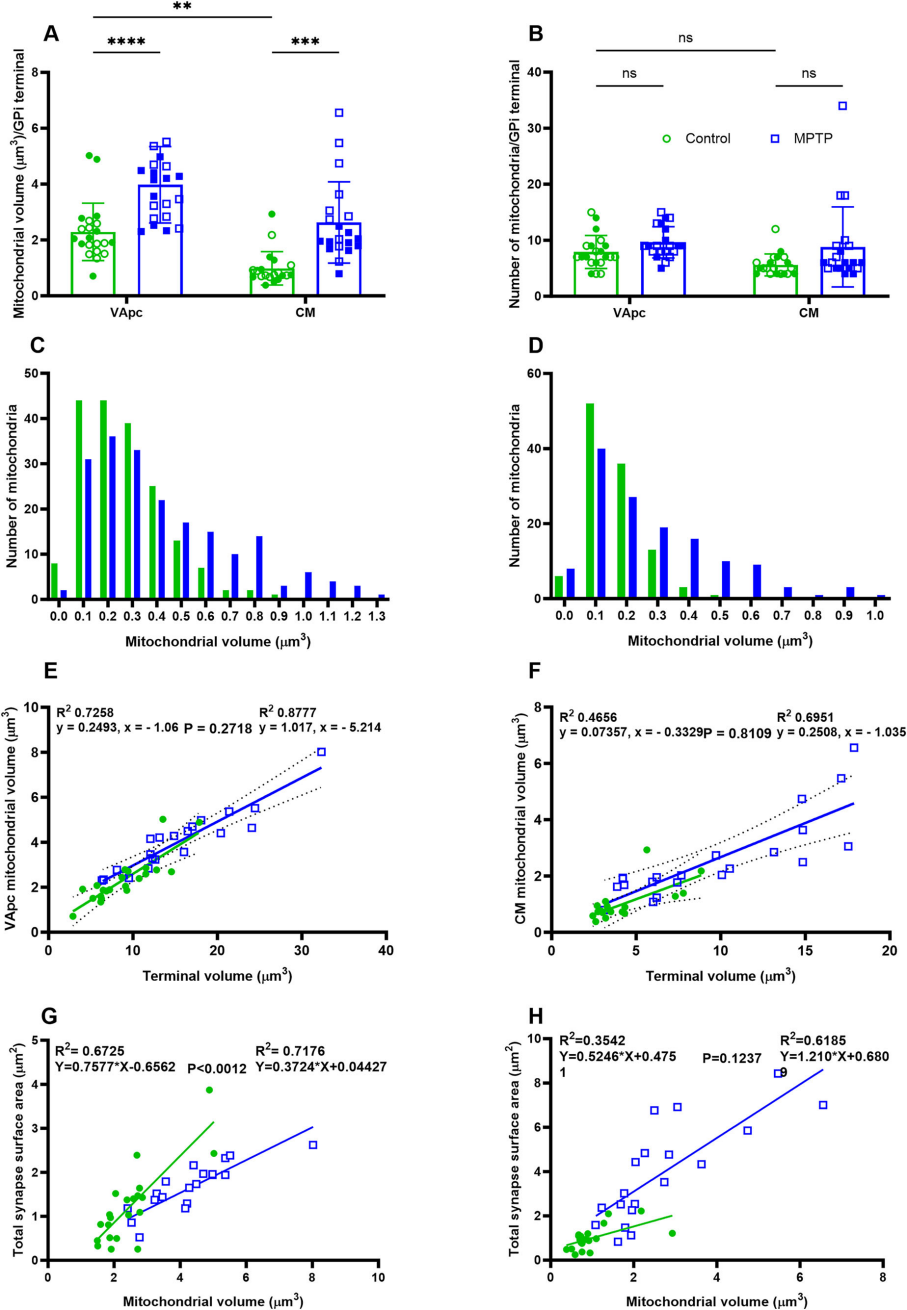
***A***, ***B***, Scatter dot plots with bar graphs comparing morphometric measurements (volume and number) of mitochondria in 3D-reconstructed GPi terminals of VApc and CM between control and parkinsonian monkeys. The mitochondrial volume per GPi terminals in the VApc and CM was significantly larger in MPTP-treated monkeys compared with that in controls (one-way ANOVA with Sidak's post hoc test; ****p < 0.0001; ***p = 0.0003; ***A***). The mitochondrial volume/GPi terminals were larger in VApc than in CM (*p = 0.0039). No significant difference was found in the number of mitochondria/GPi terminals in the VApc and CM between control and parkinsonian monkeys (***B***). ***C***, ***D***, Histograms comparing the relative frequency distribution of mitochondrial volume in GPi terminals of VApc (***C***) and CM (***D***) between control and parkinsonian monkeys. The total number of mitochondria used for control [*n* = 275 (VApc, 173, and CM = 112)] and MPTP-treated monkeys [*n* = 398 (VApc, 220, and CM: 178)]. Note the higher proportion of large mitochondria in both nuclei of parkinsonian animals. ***E***, ***F***, Scatter diagrams showing the linear regression analysis of terminal volume (μm^3^) and total mitochondria volume (μm^3^) for control (green) and MPTP-treated (blue) monkeys in VApc (***E***; *n* = 21) and CM (***F***; *n* = 20). In all cases, the terminal volume correlated positively with the total mitochondria volume (***E***,***F***). ***G***, ***H***, Scatter diagrams showing the linear regression analysis of mitochondria volume (μm^3^) and total SA (μm^2^) for control (green) and MPTP-treated (blue) monkeys in VApc (***G***; *n* = 21) and CM (***H***; *n* = 20). In all cases, the mitochondrial volume correlated positively with the total SA (***G***,***H***).

All mitochondria from 82 (41 control and 41 parkinsonian) GPi terminals analyzed in this study were fully reconstructed and morphometrically characterized. A total of 295 (173/122 in control VApc/CM) and 398 (220/178 in parkinsonian VApc/CM) mitochondria were manually traced from each image stack to generate 3D reconstructions. The data revealed that the mitochondrial volume was significantly increased in the GPi terminals in the VApc and CM (Table 3) of parkinsonian monkeys compared with that of controls (Fig. 5*A*). On average, pallidothalamic terminal mitochondria in VApc and CM were 175 and 259% larger in parkinsonian monkeys, respectively (mean volume, 3.98 µm^3^ and 2.55 µm^3^), than those in controls (mean volume, 2.26 µm^3^ and 0.98 µm^3^). However, no significant difference was found in the number of mitochondria/terminals between control and MPTP-treated monkeys (Fig. 5*B*). Mitochondrial volume heterogeneity was significant in VApc and CM (Fig. 5*A*,*C*,*D*). Frequency distribution analysis showed that ∼35% of mitochondria in GPi terminals of parkinsonian monkeys were large (volume of 0.6–1.3 µm^3^ and 0.4–1.0 µm^3^ in VApc and CM, respectively; Fig. 5*C*,*D*), while only 8% in VApc and 4% in CM were within these volume ranges in control monkeys. To determine whether the volume of mitochondria was related to the overall size of GPi terminals, we performed correlation analyses. Strong positive correlations were found between GPi terminal volumes and mitochondrial volumes (Fig. 5*E*,*F*) in both control and parkinsonian monkeys. Notably, the regression lines for the control and MPTP-treated group have no significant difference between the intercepts and slopes (Fig. 5*E*,*F*). Further, our data demonstrated a positive correlation between the mitochondrial volume and the SA of synapses in both VApc and CM of control and parkinsonian monkeys (Fig. 5*G*,*H*). The regression lines for the control and MPTP-treated group have significantly different intercepts and slopes in VApc (Fig. 5*G*), but not in CM (Fig. 5*H*).

### MCI and mitochondrial volume density between control and parkinsonian monkeys

**Figure 6.**
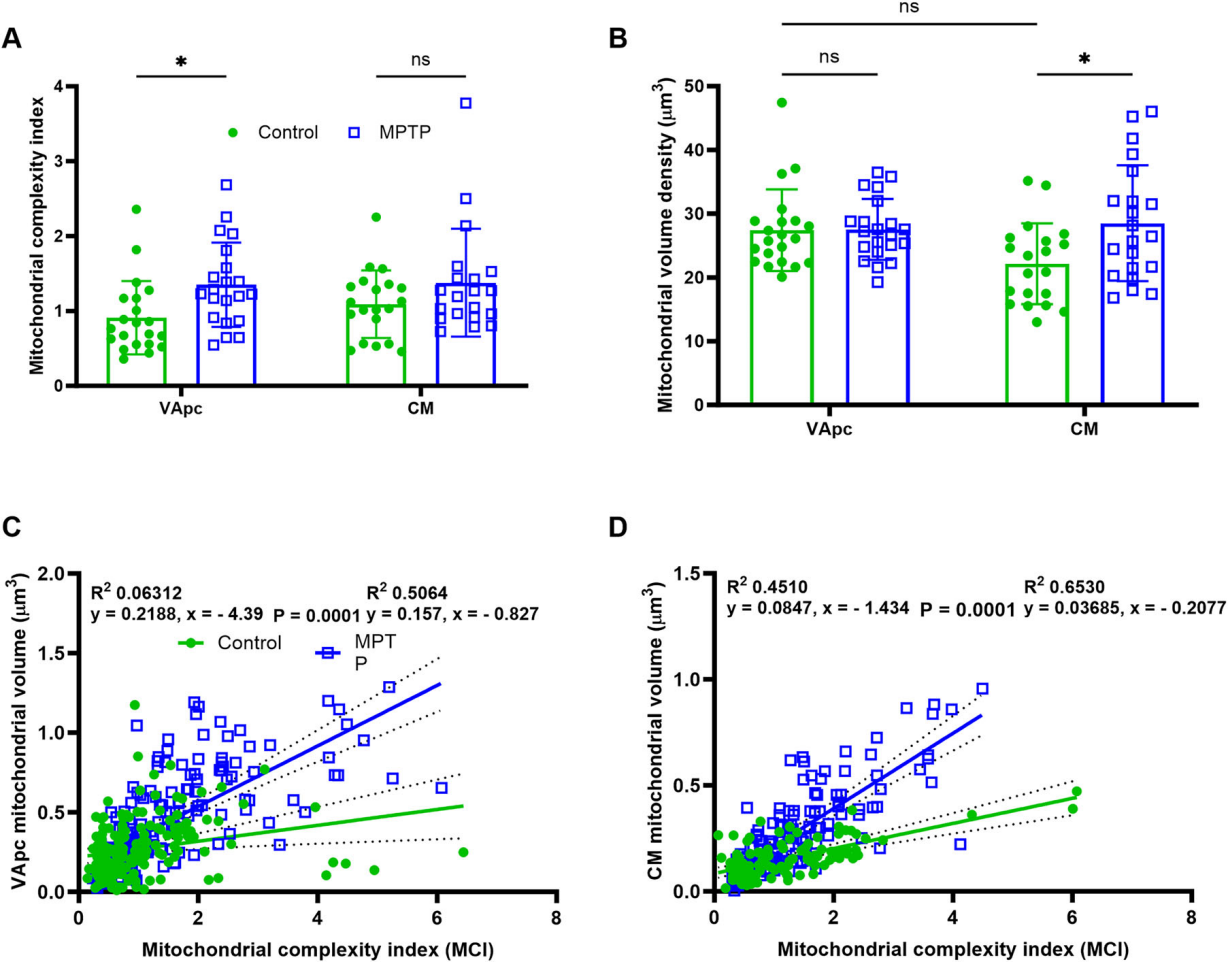
***A*, *B***, Scatter dot plots with bar graphs comparing the morphological measurements of MCI (***A***) and the MVD (mitochondrial volume normalized to terminal volume; ***B***) of mitochondria in 3D-reconstructed GPi terminals of VApc and CM between control and parkinsonian monkeys. The MCI and MVD are significantly larger in VApc and CM, respectively, of parkinsonian monkeys than those of controls (one-way ANOVA with Sidak's post hoc test; *p < 0.02; *p < 0.01). No significant difference was found in MCI and MVD in CM and VApc, respectively, between control and parkinsonian animals. ***C***, ***D***, Plotting individual mitochondrial volume and their corresponding MCI together on a mitochondrial phenotype (i.e., mitotype) graph for control (green; *n* = 186 and 112) and parkinsonian monkeys (blue; *n* = 197 and 136) in VApc (***C***) and CM (***D***), respectively, highlights significant mitochondrial morphological dissimilarities between control and parkinsonian monkeys (*p* < 0.0001).

To further assess potential changes in mitochondrial morphology of pallidothalamic terminals between the control and parkinsonian condition, we measured the MCI (Vincent et al., 2019; Faitg et al., 2021). Based on this metric, the mitochondria in GPi terminals of the VApc of parkinsonian monkeys were significantly more complex than those of controls (mean MCI, 1.351 and 0.8924; Fig. 6*A*; Table 3). However, such was not the case in CM (Fig. 6*A*, Table 3). No MCI difference was found between VApc and CM of control and parkinsonian animals (Fig. 6*A*). Next, we determined the relationships between the two variables of interest, that is, individual mitochondrial volume and its corresponding MCI, together reflecting the mitochondrial phenotype or mitotype of GPi terminals in VApc (Fig. 6*C*) and CM (Fig. 6*D*) of control and parkinsonian monkeys. The results of this regression analysis demonstrated significant dissimilarities in the mitochondrial morphology between control and MPTP-treated monkeys, in both VApc and CM (Fig. 6*C*,*D*). They also showed that the regression lines for control and MPTP-treated monkeys have significantly different intercepts and slopes (Fig. 6*C*,*D*).

To understand how MPTP-induced changes in terminal volume relate to changes in mitochondrial morphology, we measured mitochondrial volume density (MVD), defined as the percentage of terminal volume occupied by the mitochondria, in 3D-reconstructed models (Vincent et al., 2019; Faitg et al., 2021). The data suggest that there was a significant increase (30.3%; Table 3) in MVD of GPi terminals in CM, but not in VApc, of parkinsonian monkeys (Fig. 6*B*). No significant change in MVD was found between the VApc and CM of control and parkinsonian groups (Fig. 6*B*).

### Morphometric analysis and mitochondrial content of putative GABAergic nonpallidal terminals in parkinsonian monkeys

**Figure 7.**
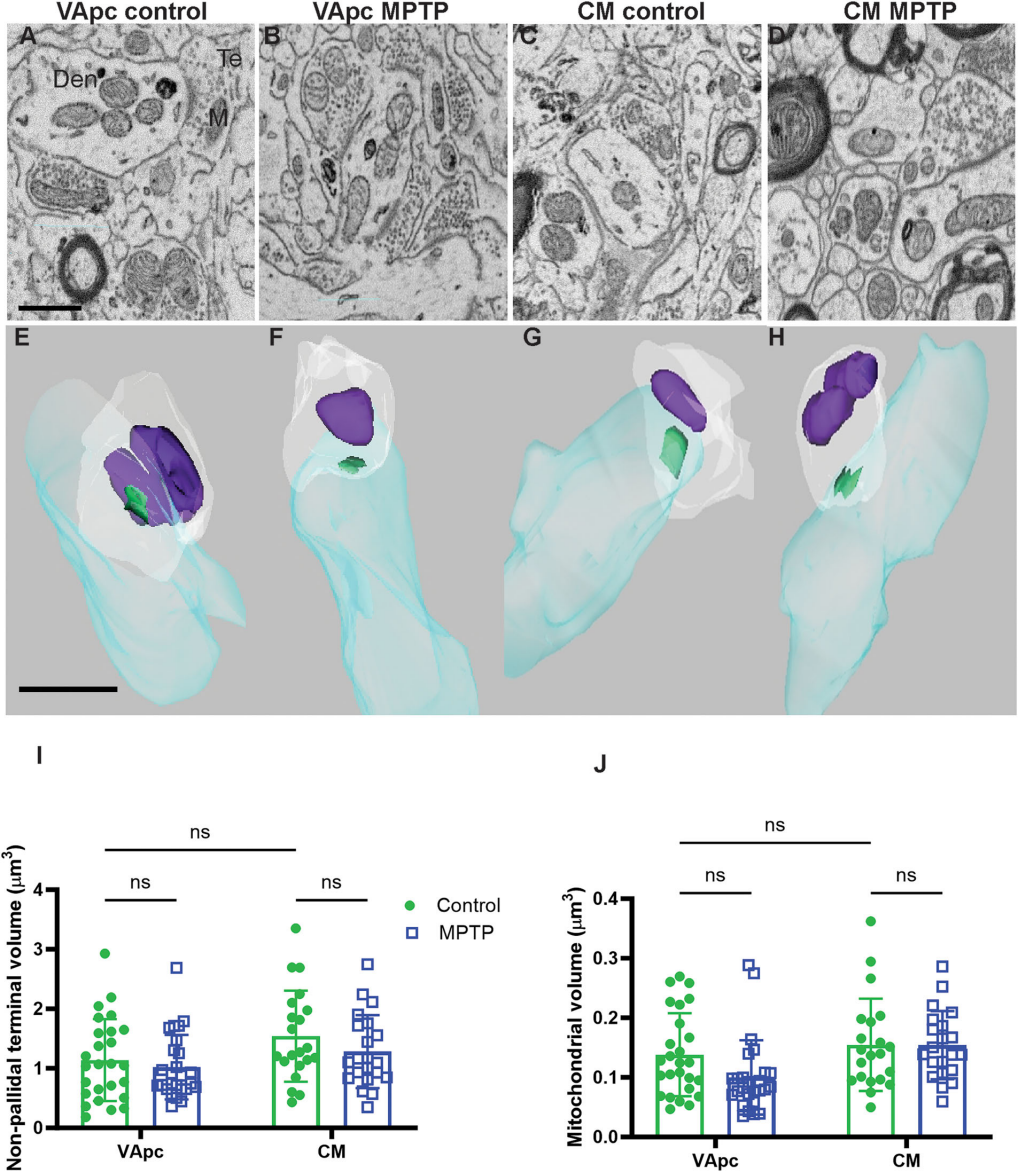
Electron micrographs (***A–D***) and reconstructed 3D models (***E–H***) of putative GABAergic nonpallidal terminals that form single symmetric axodendritic synapses in the VApc (***A***,***B***) and CM (***C***,***D***) of control and MPTP-treated parkinsonian monkeys. Den, dendrite; M, mitochondria. Scale bars: ***A***, 1 μm (valid for ***B–D***); ***E***, 1 μm (valid for ***F–H***). ***I***, ***J***, Scatter dot plot with bar graphs comparing the relative volume of nonpallidal terminals and mitochondria in the VApc and CM of control and parkinsonian monkeys. Each data point is a terminal (VApc, *n* = 26, and CM, *n* = 21). Statistical differences were determined by two-way ANOVA for repeated measures followed by the Sidak post hoc test. No ultrastructural differences were noticed between putative GABAergic nonpallidal terminals and their mitochondria in VApc and CM of control versus MPTP-treated monkeys.

To determine whether the morphological and mitochondrial changes found in GPi terminals were also seen in other GABAergic terminals in VApc and CM, we reconstructed and analyzed the morphology and mitochondrial content of 90 (22/21 in control VApc/CM and 26/21 parkinsonian VApc/CM) putative GABAergic nonpallidal terminals in the VApc and CM of parkinsonian and control monkeys (Fig. 7*A–H*). From these terminals, a total of 135 mitochondria (27/40 in control VApc/CM and 34/34 in parkinsonian VApc/CM) were manually traced from each image stack and reconstructed. As expected, these 3D-reconstructed nonpallidal terminals were much smaller in size and displayed a lower mitochondrial volume than GPi terminals in VApc and CM (compare Figs. 3*I*,*J*, 5*A* with Fig. 7*I*,*J*). In contrast to GPi terminals, no significant difference in terminal and mitochondrial volume was found in these putative GABAergic nonpallidal terminals of VApc and CM between the control and parkinsonian monkeys (Fig. 7*I*,*J*; Table 3).

## Discussion

In this study, we used the SBF-SEM 3D electron microscopy approach to assess ultrastructural changes in the morphometry, synaptic connections, and mitochondrial content of GPi terminals in the basal ganglia-receiving regions of the ventral motor thalamus and the CM of MPTP-treated parkinsonian monkeys. Two main conclusions can be drawn from our observations: (1) GABAergic pallidothalamic, but not putative nonpallidal, terminals undergo major ultrastructural changes in the VApc and CM of parkinsonian monkeys and (2) GPi terminals in VApc and CM display different ultrastructural features, suggesting that the morphology and, most likely, the synaptic properties of pallidothalamic terminals originating from single pallidal axons differ between VApc and CM. These findings suggest a potential contribution of disrupted structure–function relationships of the pallidothalamic system in parkinsonism.

### Pallidothalamic terminals in the primate VApc and CM

In keeping with previous studies (Parent and De Bellefeuille, 1983; Ilinsky and Kultas-Ilinsky, 1987; Rouiller et al., 1994; Sakai et al., 1996), injections of AAV5-EYFP in the ventrolateral GPi resulted in dense anterograde labeling in the sectors of the VA/VL and CM known as the source of projections to motor cortices and the sensorimotor striatum (Matelli et al., 1989; Darian-Smith et al., 1990; Inase and Tanji, 1995; McFarland and Haber, 2002; Sidibe et al., 2002; Smith et al., 2014). Consistent with the previous literature (Kultas-Ilinsky et al., 1983; Sidibe et al., 1997; Rovo et al., 2012; Swain et al., 2020), our 3D EM reconstruction data demonstrated that pallidothalamic terminals were large (1.0–3.0 μm in diameter), densely filled with mitochondria, and formed multiple axodendritic synapses in the VApc and CM of rhesus monkeys. Despite some variability, the 3D volumetric data and number of synapses of pallidothalamic terminals in VApc reported in this study are in line with those described for nigrothalamic terminals in the monkey magnocellular VA (VAmc; Bodor et al., 2008), corroborating evidence that both GPi and SNr give rise to large multisynaptic GABAergic terminals in the primate ventral motor thalamus (Kultas-Ilinsky and Ilinsky, 1990; Ilinsky et al., 1997). Other large, multisite, GPi-like, GABAergic terminals from the zona incerta (Bartho et al., 2007) and the anterior pretectal nucleus (APT) exert strong inhibitory effects upon their postsynaptic thalamic targets (Bartho et al., 2002; Halassa and Acsady, 2016). For instance, activation of APT terminals generates a larger charge transfer and greater persistent current, even at high stimulation frequencies, in thalamocortical cells (Wanaverbecq et al., 2008) than the stimulation of the monosynaptic GABAergic RTN terminals (Wanaverbecq et al., 2008), thereby suggesting that the morphology and synaptic architecture or GABAergic pallidothalamic afferents may dictate their strength and neuroplastic properties in normal and parkinsonian conditions (see details below). It is noteworthy that the GABAergic terminals from GPi and SNr are largely segregated in the ventral motor nuclei (VApc vs VAmc) and caudal intralaminar nuclei (CM vs Pf), and both are separated from the pretectal and incertal GABAergic terminals that are mainly confined to posterior high-order thalamic regions (Kultas-Ilinsky and Ilinsky, 1990; Ilinsky et al., 1997; Sidibe et al., 2002; Bartho et al., 2007; Wanaverbecq et al., 2008). Given this segregation, we are confident that the unlabeled multisynaptic terminals reconstructed from the VApc in the present study largely originate from the GPi.

### Ultrastructural differences between GPi terminals in VApc and CM: potential significance and regulatory mechanisms

The present 3D volumetric analysis confirmed that GPi terminals in the VApc and CM display characteristically different ultrastructural features in control monkeys (Balercia et al., 1996; Sidibe et al., 1997). Knowing that pallidothalamic projections to the VApc and CM mainly originate from single GPi neurons (Parent and De Bellefeuille, 1983; Parent and Hazrati, 1995), these observations suggest target-specific ultrastructural differences in the morphometry of GPi terminals between the VApc and CM in primates. Whether such anatomical differences are reflected in the neuroplastic properties and strength of pallidothalamic synapses that impinge upon CM versus VApc neurons in normal and diseased states remains to be established. Given the previous data showing that differences in morphology and synaptic arrangement of various populations of GABAergic terminals are associated with contrasting functional properties in other thalamic nuclei (Wanaverbecq et al., 2008), future studies that compare the synaptic properties of GPi inputs to VApc and CM are needed to further characterize the structure–function relationships of nuclei-specific pallidothalamic terminals in the primate motor thalamus. Another example of single axons that give rise to structurally (and likely functionally) different terminals in their projection targets are those from layer V pyramidal tract neurons that send collateralized projections to high-order posterior thalamic nuclei (lateroposterior nucleus and pulvinar) and the striatum (Pare and Smith, 1996). The data from this study and others suggested that the morphology of corticothalamic and corticostriatal terminals that originate from single pyramidal tract axons was strikingly different (Kemp and Powell, 1971; Pare and Smith, 1996; Raju et al., 2008; Villalba and Smith, 2011), such that corticothalamic boutons were large and formed multiple synapses predominantly with dendrites of GABAergic interneurons (Pare and Smith, 1996), while corticostriatal terminals are much smaller in size and largely form single axospinous synapses (Raju et al., 2008; Villalba and Smith, 2011). Given that the development and maturation of synapses is complex and involves a myriad of temporally sequenced physiological and molecular mechanisms (Andreae and Burrone, 2018; Favuzzi and Rico, 2018), it is possible that specific target-derived chemical or physiological cues may regulate the development of individual terminals that originate from single GPi axons in VApc versus CM. In a recent study, Hayashi et al. (2021) showed that the multisynaptic arrangement of giant layer V corticothalamic terminals in the posterior nucleus (Sherman and Guillery, 2011; Rovo et al., 2012) is dependent on the presynaptic control of the regulated vesicular release by the synaptosome-associated protein 25 (SNAP25). Whether SNAP25 or other regulators of synaptic development are involved in the maturation of pallidothalamic boutons in CM and VApc remains to be established. There is also evidence for nuclei-specific differences in morphology of retinal inputs to various nuclei of the visual thalamus, but it is not clear if these terminals originate from single or multiple retinal ganglion cells (Hammer et al., 2014). The recent introduction of retrograde viral vectors that label extensively the axon collaterals of retrogradely transduced neurons should help advance knowledge in this field (Tervo et al., 2016; Albaugh et al., 2020; Galvan et al., 2023; Kim et al., 2023).

### Ultrastructural remodeling of pallidothalamic terminals in parkinsonian state: functional significance

Our findings demonstrate that the volume as well as the number and size of synapses formed by pallidothalamic terminals in VApc and CM undergo robust ultrastructural changes in MPTP-treated parkinsonian monkeys, whereas the morphology of putative nonpallidal GABAergic terminals remains intact under these conditions. Given the converging evidence that the large terminal volume and multisynaptic connections have an impact upon neurotransmitter release, synaptic strength, and synaptic plasticity in the mammalian thalamus (Bodor et al., 2008; Nishijima et al., 2020), it is tempting to speculate that the increased volume of GPi terminals in parkinsonian monkeys allows for the formation of a greater number of synapses and/or synapses of a greater area, which could help increasing the tonic GABAergic tone of pallidal terminals upon VApc and CM neurons in parkinsonism.

Although it is well established that the firing rate and bursting pattern of GPi neurons are increased in moderate parkinsonian monkeys (Wichmann et al., 1999; Soares et al., 2004; Hahn et al., 2008; Muralidharan et al., 2016) and PD patients (Hutchison et al., 1994), the impact of this abnormal BG GABAergic output on motor thalamic firing rates in animal models of parkinsonism or PD patients remains unclear, some studies providing evidence for decreased neuronal firing (Vitek et al., 1994; Schneider and Rothblat, 1996; Ni et al., 2000; Molnar et al., 2005; Chen et al., 2010), while others found no change (Pessiglione et al., 2005) or even increase in firing rates (Zirh et al., 1998; Magnin et al., 2000; Guehl et al., 2003; Bosch-Bouju et al., 2014). Furthermore, studies of MPTP-treated monkeys suggest an increased metabolic activity in the ventral motor thalamus (Mitchell et al., 1989; Rolland et al., 2007), possibly reflecting the increased activity of basal ganglia inputs. Altogether, these findings indicate that the basal ganglia-mediated GABAergic regulation of the thalamocortical system is disrupted and that these changes likely contribute to the pathophysiology of the basal ganglia–thalamocortical loops in PD. Although they do not demonstrate a causal relationship, our findings suggest that neuroplastic changes in the synaptic organization of pallidothalamic terminals may contribute to these pathophysiological effects. An important issue to investigate is whether these ultrastructural changes are associated with the development and severity of parkinsonism. An assessment of the anatomofunctional integrity of the pallidothalamic system during the course of nigrostriatal dopamine denervation in the chronic MPTP-treated monkey model of PD (Masilamoni and Smith, 2018) is warranted to further address these issues.

### Mitochondrial morphology alterations in the pallidothalamic terminals of parkinsonian monkeys

Mitochondria are critically important for proper synaptic function, due to their central role in ATP production, Ca^2+^ regulation, and other major signaling mechanisms. Presynaptic functions including neuronal activity and synaptic strength rely directly on mitochondria-driven ATP synthesis (Gulyas et al., 2006; Kann et al., 2014). Given that the demand for mitochondrial function is reasonably coupled to neuronal activity (Gulyas et al., 2006; Kann et al., 2014) and directly correlated with synaptic strength (Verstreken et al., 2005; Ivannikov et al., 2013; Sun et al., 2013; Smith et al., 2016), our data suggest that GPi terminals in the VApc might have higher energetic demands than in CM (Justs et al., 2022). Furthermore, our 3D EM data demonstrate that there is a significant correlation between increases in mitochondrial volume, terminal volume, and synapses SA in both the VApc and CM of MPTP-treated parkinsonian monkeys. These observations suggest that the ultrastructure and the composition of presynaptic mitochondria might be associated with synaptic performance in both control and parkinsonian monkeys. Numerous studies have, indeed, demonstrated that mitochondrial volume changes correlate with firing rate, mitochondrial Ca^2+^ uptake, and synaptic vesicle number in different brain regions (Gulyas et al., 2006; Ivannikov et al., 2013; Cserep et al., 2018; Lewis et al., 2018; Rodriguez-Moreno et al., 2018; Thomas et al., 2019).

Various anomalies in mitochondrial volume and cristae have been reported in PD patients (Trimmer et al., 2000) and MPTP-treated animal models (Tanaka et al., 1988; Mizukawa et al., 1990; Levi et al., 1994). Although we did not notice any mitochondrial inclusions or disordered mitochondrial cristae in pallidal terminals that innervate VApc and CM of parkinsonian monkeys, significant differences in the mitochondrial morphology and the mitotype correlation graph of pallidothalamic terminals were found between control and MPTP-treated monkeys. Higher MCI in VApc indicates that the mitochondrial shape has a more complex and higher SA relative to the volume (Brown et al., 2022). This eventually leads to increased O_2_ consumption and oxidative phosphorylation. A similar phenotype of increased mitochondrial complexity with aging was also observed in aging mouse skeletal muscle (Leduc-Gaudet et al., 2015), postulated to reflect compensatory hyperfusion response to stress, previously reported in cellular systems (Shutt and McBride, 2013). In contrast, the MVD increase in the pallidothalamic terminals in CM of parkinsonian monkeys might reflect mitochondrial abundance to increase ATP production and meet energy demand necessary to maintain the tonic increase in pallidothalamic outflow (Bereiter-Hahn and Voth, 1994; Prakash et al., 2017).

### Conclusions

Altogether, results of this study demonstrate that GABAergic pallidothalamic terminals are endowed with a high level of structural plasticity that may contribute to the development and maintenance of the abnormal increase in pallidal outflow to the thalamus in the parkinsonian state. Furthermore, the evidence for ultrastructural differences between GPi terminals in VApc and CM suggests that morphologically distinct pallidothalamic terminals may underlie specific physiological properties of pallidal inputs to VApc or CM neurons in normal and diseased states. The present data lays the foundation for future electrophysiological studies that will examine transmitter dynamics and postsynaptic responses to eventually elucidate the functional consequences of ultrastructural changes in terminal volume and the number and size of synapses of pallidothalamic terminals in PD pathophysiology.

## Synthesis

Reviewing Editor: Mikko Airavaara, Helsingin yliopisto - Viikin kampus Decisions are customarily a result of the Reviewing Editor and the peer reviewers coming together and discussing their recommendations until a consensus is reached. When revisions are invited, a fact-based synthesis statement explaining their decision and outlining what is needed to prepare a revision will be listed below. The following reviewer(s) agreed to reveal their identity: Susan Sesack, Laszlo Acsady. Note: If this manuscript was transferred from JNeurosci and a decision was made to accept the manuscript without peer review, a brief statement to this effect will instead be what is listed below.

This paper describes the work of Masilamoni and collaborators in the lab of Yoland Smith, a leading expert in the MPTP primate model of PD. The research reports alterations to axon terminals from the GPi synapsing in the VApc and CM thalamus of control and experimental monkeys. The latter underwent MPTP-induced loss of dopaminergic inputs to the basal ganglia to produce chronically developing moderate parkinsonian symptoms. Some animals had viral tract-tracing to verify GPi to thalamus projections. The team then sacrificed the monkeys, fixed the brain tissue, and used serial block face scanning electron microscopy with reconstruction to investigate pallidothalamic axons: their size, synaptic features, and mitochondrial content. The authors report major morphological changes in this axon terminal group that were not seen in another inhibitory population of axons intrinsic to thalamus. The results have important implications for understanding pathophysiological changes in the activity of motor thalamic regions in parkinsonian animal models and PD in humans. The paper is well written and well supported by data from state-of-the-art quantitative methods. There are, however, several questions about the technical approaches, results, and interpretations that, once addressed, will lend greater clarity to a fine study.

1. Basal ganglia terminals are linked to postsynaptic dendrites via two types of contacts, the more peripherally located synapses and the more centrally located puncta adherentia as clearly shown by Ilinsky et al. 1997, Kultas-Ilinksy et al. 1997 and Bodor et al., 2008, all properly cited by the authors. Vesicle accumulation is evident in the first but lacking in the second type of contact. According to our present understanding of neurons synaptic transmission takes place at the synapses but not at the puncta. The present ms does not differentiate between these two. There are several ways to overcome this problem, but the authors need to somehow address it and state whether it is possible or not to disambiguate these two types of contacts in this material.

E.g a brief series of 3-4 EM image may help to clarify this point.

2. The main rationale for this study is that the firing rate of GPi neurons increase and switch to a burst mode during Parkinson's. While the authors have assessed the monkeys to have moderate Parkinson's based on motor phenotype, there is no confirmation that the GPi neurons have switched to the "increased and burst" firing mode at this time. Analysis of other downstream motor targets such as the ventral lateral nucleus will add more depth to the data.

3. When assessing monkey motor skill, was there any possibility for the expert rater to at least begin in a blinded fashion? As symptoms progressed, one assumes the differences between animals became more overt such that blinding was not achievable. In the manuscript clearly state how blinding and randomization were carried out.

4. Two different tracers were delivered via two rather different methods. The authors should state whether any of these differences contributed to the outcomes of the study. Also, why was it necessary to use physiology to define the GPi in MPTP monkeys but not controls? This was not apparent.

5. Please provide the source(s) of primary anti-GFP and secondary antibodies. Also, it would be a kindness to readers unfamiliar with fluorescent proteins to indicate that antibodies against GFP will also recognize EYFP, the actual tracer in these experiments. Double check that all the RRIDs are present in the methods part.

6. Renovo appears suddenly in the methods without prior use. Its only other appearance in the text is in the Acknowledgements. For clarity, please explain that Renovo is part of the Cleveland Clinic group providing the SBF-SEM service (if that is correct).

7. The authors state they chose "labeled and unlabeled GPi terminals...at random" and then explain how they employed ultrastructural criteria to identify unlabeled GPi axons forming synapses. However, assuming I understood the background materials correctly, two references cited in the paper indicate that nigrothalamic axon terminals and inputs from the anterior pretectal area also have large terminals forming multiple synapses like those of the GPi (Bodor et al., 2008 and Wanaverbecq et al., 2008). The latter information is also in the discussion. Hence, even if the authors confirmed using tracing that GPi axons and synapses are of the type described, how can they be certain there is absolute identity between every axon forming that type of synapse and a GPi termination? This seems quite important to the conclusions of the study.

8. The authors state (page 12) that pallidothalamic terminals form synapses "with projection neurons." Is it certain the postsynaptic targets are projection neurons versus local circuit neurons? The latter have been described in the thalamus, especially in primates. Are they clearly absent from the VApc and CM, making it safe to assume projection neurons are the only possible targets?

9. The style of the results is difficult to follow precisely. Sometimes quantitative data is provided without the statistical comparison, whereas the opposite also happens, i.e. the statistics are presented without the quantitative data. Much of the relevant information is in the figures and figure legends, and so the authors should streamline the text here by being consistent in their style of presentation. Statistical table is highly recommended in the eNeuro instructions. See example:

https://www.eneuro.org/content/eneuro/7/2/ENEURO.0218-19.2020/DC1/embed/inline-supplementary-material-1.docx?download=true

10. In describing the regression analysis for Figure 2C,D (page 13), the authors indicate significant differences in the VApc, "but not so much in CM (Figure 2D, P=0.0208)." Given that the P value is still significant, it might be better to say "and less so in CM."

11. The data in Figure 1 ignores the contributions of individual animals. It would be useful to reveal whether any individual monkey drove the outcomes. This could be accomplished by using two differently colored dots (or squares) to denote the two individuals within each group of 20 observations.

12. Incidentally, both VApc groups show 21 data entries in Figure 1; why is there one extra? For Figure 3, mitochondrial volume, the control VApc has 22 data entries, as does this group for Figure 4, mitochondrial complexity index. Figure 5I VApc control has 22 data points for RT terminal volume and 26 data points for mitochondrial volume. Please check all the graphs carefully, as I quit counting the dots!

13. The legends for Figures 3 and 4 indicate that panels A and B are histograms, whereas they are bar graphs showing individual data points. Only panels C and D for Figure 3 show histograms. Also, the legend repeats part of the description of C-D panels.

14. The legend for Figure 4 provides the formula for calculating the mitochondrial complexity index, but the superscripts are missing. Please correct.

15. The part of the discussion dedicated to regional differences between the VApc and CM, even in control animals, was somewhat disappointing, given such interesting findings. Clearly much "remains to be established," but is there truly nothing in the literature at present that the observed distinctions might relate to? They are especially interesting given that the same GPi neuron produces both types of axon terminals and that they likely differ as well in their mitochondrial capacity (present study). Is anything known about these parts of thalamus that might indicate local factors affecting the structure and function of extrinsically derived axons? On a related note, are the authors aware of any other reports of mitochondrial differences between collateralized axons known to originate from the same cells? The authors only cite papers describing variance across brain regions.

16. Please consistently label synapses with arrows in all EM images.

17. Add a light microscopic image of the terminals and a drawing depicting the exact position of the EM samples shown in a stereotactic map indicating the AP level.

18. The legend of Fig. 2. reads as follows: "The number of synapses and surface area per GPi axon terminals are significantly larger in parkinsonian monkeys than in controls (one-way ANOVA with Tukey's post hoc test, ***P = 0.0005; ****P < 0.0001)." A reference to CM is missing from this sentence.

19. When the complexity of the pallidal terminals is discussed, a reference to Bodor et al. ,2008 might be considered since this is the first study which reconstructed basal ganglia terminals of primates in 3D. The exact numerical values (no. of synapses, size of terminals correlation between the two) of the two studies can be compared.

20. A brief description of mitochondrial complexity index may be appropriate even if a reference is provided to illustrate the changes in mitochondria in representative EM images.

21. RTN-like terminals. A very nice addition to the study is that a group of non-pallidal terminals are shown which does not change their morphology. However, the authors refer to these terminals as GABAergic terminals, but GABA labelling has not been performed. Based on the images provided it is not entirely convincing that purely based on the structure of the postsynaptic density the identity of this group of terminals can be determined. In Fig 5 B,C,D it is not labelled which boutons the authors consider as RTN-like. My suggestion is that either show convincing images about asymmetric and symmetric terminals or use the term "non-pallidal" rather than RTN-like. A brief series of 3-4 EM image may help to clarify this point as well.

22. Please state in the ms that these reconstructed non-pallidal terminals did not receive afferent inputs thus are unlikely belong to F2 terminals.

23. The authors employ a synapse-based approach, treating each synapse as an independent data point for statistical analysis. This method artificially inflates the sample size (n) compared to a methodology that would involve averaging data across individual animals. This should be discussed and the authors should clearly state what is their n, and how they have justified it. Also, discussion on the matter is needed.

24. There is a lack of functional output on how increase in synapse volume or an increase in mitochondrial complexity index impacts synaptic activity at GPi terminals. Either a mitochondrial activity assay or recordings at these synapses would truly elevate the paper and help understand how these structural changes impact synapse function or more discussion on this matter is needed.

25. The assertion regarding structural plasticity in GPi terminals based on target specificity is interesting but needs to further developed to understand the significance of this finding.

26. visual abstract are missing

## Author Response

January 17, 2024 Dear Editors Thanks for giving us the opportunity to submit a revised version of our manuscript entitled "Structural Plasticity of GABAergic Pallidothalamic Terminals in MPTP-treated Parkinsonian Monkeys: A 3D Electron Microscopic Analysis" for publication in eNeuro. We are grateful to the reviewer for the thoughtful comments which, we believe, helped us strengthened the foundation of our article. Please, find below a detailed account of the changes we have made to the paper to address the reviewer's suggestions. In this report, the reviewer's comments are printed in black and our responses are in blue.

Reviewer's rebuttal This paper describes the work of Masilamoni and collaborators in the lab of Yoland Smith, a leading expert in the MPTP primate model of PD. The research reports alterations to axon terminals from the GPi synapsing in the VApc and CM thalamus of control and experimental monkeys. The latter underwent MPTP-induced loss of dopaminergic inputs to the basal ganglia to produce chronically developing moderate parkinsonian symptoms. Some animals had viral tract-tracing to verify GPi to thalamus projections. The team then sacrificed the monkeys, fixed the brain tissue, and used serial block face scanning electron microscopy with reconstruction to investigate pallidothalamic axons: their size, synaptic features, and mitochondrial content. The authors report major morphological changes in this axon terminal group that were not seen in another inhibitory population of axons intrinsic to thalamus. The results have important implications for understanding pathophysiological changes in the activity of motor thalamic regions in parkinsonian animal models and PD in humans. The paper is well written and well supported by data from state-of-the-art quantitative methods. There are, however, several questions about the technical approaches, results, and interpretations that, once addressed, will lend greater clarity to a fine study.

We thank the reviewer for the appreciation and criticisms of our manuscript. We have taken into consideration all suggestions made by the reviewer to increase the overall quality of the paper.

1. Basal ganglia terminals are linked to postsynaptic dendrites via two types of contacts, the more peripherally located synapses and the more centrally located puncta adherentia as clearly shown by Ilinsky et al. 1997, Kultas-Ilinksy et al. 1997 and Bodor et al., 2008, all properly cited by the authors. Vesicle accumulation is evident in the first but lacking in the second type of contact. According to our present understanding of neurons synaptic transmission takes place at the synapses but not at the puncta. The present ms does not differentiate between these two. There are several ways to overcome this problem, but the authors need to somehow address it and state whether it is possible or not to disambiguate these two types of contacts in this material.

E.g a brief series of 3-4 EM image may help to clarify this point.

We thank the reviewer for this excellent suggestion. As per the reviewer's suggestion, we added 4-5 serial EM images of a single terminal in the figure 2, and clearly state what ultrastructural criteria we used to differentiate synapses from puncta in the revised manuscript (page 10).

2. The main rationale for this study is that the firing rate of GPi neurons increase and switch to a burst mode during Parkinson's. While the authors have assessed the monkeys to have moderate Parkinson's based on motor phenotype, there is no confirmation that the GPi neurons have switched to the "increased and burst" firing mode at this time. Analysis of other downstream motor targets such as the ventral lateral nucleus will add more depth to the data.

The reviewer is correct. We did not record activity of GPi neurons in the monkeys used in this study, thus cannot say for sure that the pallidothalamic terminals examined originated from neurons that displayed burst firing. However, we cite data from the published literature which provide evidence that burst firing is very commonly seen in the GPi of MPTP-treated parkinsonian monkeys and PD patients which, we believe, is a foundation strong enough to suggest that burst firing was likely to be the pattern of activity GPi neurons displayed in the parkinsonian monkeys used in this study (see page 20).

3. When assessing monkey motor skill, was there any possibility for the expert rater to at least begin in a blinded fashion? As symptoms progressed, one assumes the differences between animals became more overt such that blinding was not achievable. In the manuscript clearly state how blinding and randomization were carried out.

The reviewer raises an important criticism about blinded motor skill assessments. Each monkey video collected during the course of MPTP administration was, indeed, scored by two investigators, including one who was blinded to the status of the animal's MPTP treatment The differences in the rating scores between the two observers for monkeys used in the present study were <6%. The mean values obtained by the two experimenters were used for the study. The information is included in the revised manuscript (page 6).

4. Two different tracers were delivered via two rather different methods. The authors should state whether any of these differences contributed to the outcomes of the study. Also, why was it necessary to use physiology to define the GPi in MPTP monkeys but not controls? This was not apparent.

We are sorry for the lack of explanation as to why different approaches were used to deliver the AAV5 in the GPi of control versus MPTP-treated monkeys. The reason why we used in vivo recordings to target GPi in the two MPTP monkeys is because these animals had recording chambers fixed on their head to collect data for an in vivo electrophysiology experiment unrelated to the present study prior to the injections of AAV5 in the GPi. In brief, once the collection of electrophysiology data was completed, we used in vivo recording to map the border of the GPi and deliver AAV5 through an injectrode. In both control and MPTP-treated monkeys, the viral vector injected was the same (AAV5-hsyn-eYFP) except for the ChR2 vs Arch3 transgenes. To our knowledge, there is no evidence that the expression of either of these channel rhodopsins interfere with the anterograde transport properties of AAV5. In fact, the extent of anterograde transduction of pallidothalamic terminals achieved with both vectors was comparable in the different animals used in our study, irrespective of the vector used. It is also important to note that the main use of these vectors in the present study was merely to identify some GPi terminals without any attempt at comparing the relative abundance of terminal labeling between individual cases. The information is included in the revised manuscript (page 7).

5. Please provide the source(s) of primary anti-GFP and secondary antibodies. Also, it would be a kindness to readers unfamiliar with fluorescent proteins to indicate that antibodies against GFP will also recognize EYFP, the actual tracer in these experiments. Double check that all the RRIDs are present in the methods part.

As per the reviewer suggestion, we have included the primary and secondary antibody information, including RRDIs, in Table 2. We have also added a note indicating that the GFP antibody recognizes EYFP. These details are presented in the Methods section of the revised submission (page 8).

6. Renovo appears suddenly in the methods without prior use. Its only other appearance in the text is in the Acknowledgements. For clarity, please explain that Renovo is part of the Cleveland Clinic group providing the SBF-SEM service (if that is correct).

We are sorry for this oversight. Because the original name of the company was Renovo when we started collaborating with them, we forgot to replace it by their new name which is now Cleveland Clinic group. This information has been clarified in the revised manuscript (page 10, 11).

7. The authors state they chose "labeled and unlabeled GPi terminals...at random" and then explain how they employed ultrastructural criteria to identify unlabeled GPi axons forming synapses. However, assuming I understood the background materials correctly, two references cited in the paper indicate that nigrothalamic axon terminals and inputs from the anterior pretectal area also have large terminals forming multiple synapses like those of the GPi (Bodor et al., 2008 and Wanaverbecq et al., 2008). The latter information is also in the discussion. Hence, even if the authors confirmed using tracing that GPi axons and synapses are of the type described, how can they be certain there is absolute identity between every axon forming that type of synapse and a GPi termination? This seems quite important to the conclusions of the study.

This is an important point. To avoid confusion between GPi terminals and other ultrastructurally similar boutons in our analysis, we selected blocks of tissue that were strictly confined to the VApc, the GPi-receiving region of the VA/VL nuclear group. Given that pallidal and nigral inputs are largely segregated in the primate motor thalamus (Ilinsky et al., 1985, JCN 236:315), and that both basal ganglia afferents do not overlap with the projections from the anterior pretectal region in the posterior thalamus (Wanaverbecq et al., 2008), we are confident that the multisynaptic GABAergic terminals examined in the VApc are from the GPi. Similarly, multisynaptic inputs to the CM originate mainly from GPi, whereas nigral inputs to the caudal intralaminar complex are confined to the parafascicular nucleus (Sidibe et al. 1997, JCN 382:323; Sidibe et al., 2002, JCN 447:286). We have clarified these points in the revised manuscript (page 18).

8. The authors state (page 12) that pallidothalamic terminals form synapses "with projection neurons." Is it certain the postsynaptic targets are projection neurons versus local circuit neurons? The latter have been described in the thalamus, especially in primates. Are they clearly absent from the VApc and CM, making it safe to assume projection neurons are the only possible targets? The reviewer is correct. The primate thalamus, including the VApc and CM regions analyzed in our study, contain both projection neurons and GABAergic interneurons (Smith et al., 1987; Neuroscience 22:579). In the present study, we differentiated dendrites of GABAergic interneurons vs projection neurons based on the presence or not of synaptic vesicles and the formation or not of dendro-dendritic synapses, which are known ultrastructural features of thalamic GABAergic interneuron dendrites in the primate motor thalamus (Ilisnky et al., 1999, JCN 409:369). Thus, based on these structural features, we feel confident that we can categorize dendrites as belonging to projection neurons or interneurons. This issue has been clarified in the revised manuscript (page 12).

9. The style of the results is difficult to follow precisely. Sometimes quantitative data is provided without the statistical comparison, whereas the opposite also happens, i.e. the statistics are presented without the quantitative data. Much of the relevant information is in the figures and figure legends, and so the authors should streamline the text here by being consistent in their style of presentation. Statistical table is highly recommended in the eNeuro instructions. See example: https://www.eneuro.org/content/eneuro/7/2/ENEURO.0218-19.2020/DC1/embed/inline-supplementary-material-1.docx?download=true.

We appreciate the reviewer's comments. As per the reviewer suggestions the style of results has been modified throughout the results section and a statistical table is now included in the revised manuscript.

10. In describing the regression analysis for Figure 2C,D (page 13), the authors indicate significant differences in the VApc, "but not so much in CM (Figure 2D, P=0.0208)." Given that the P value is still significant, it might be better to say "and less so in CM." We have modified the text as per reviewer suggestion. Now the Figure number is 4C, D in the revised manuscript 11. The data in Figure 1 ignores the contributions of individual animals. It would be useful to reveal whether any individual monkey drove the outcomes. This could be accomplished by using two differently colored dots (or squares) to denote the two individuals within each group of 20 observations.

Thanks to the reviewer for this excellent suggestion. In line with the recommendation, we have modified the figure (in the revised manuscript it is figure 3) using two differently colored dots and performed t-test to see any variations with the test groups. Results show that there is no significant difference between data collected from the two control animals in both nuclei and for the VApc in the 2 MPTP-treated monkeys, but a significant inter-individual difference was found for data gathered from the CM in the MPTP group. This information was added to the Methods sections (page 11).

12. Incidentally, both VApc groups show 21 data entries in Figure 1; why is there one extra? For Figure 3, mitochondrial volume, the control VApc has 22 data entries, as does this group for Figure 4, mitochondrial complexity index. Figure 5I VApc control has 22 data points for RT terminal volume and 26 data points for mitochondrial volume. Please check all the graphs carefully, as I quit counting the dots! Sorry for the confusion about the total number of terminals analyzed in each data set. Although the goal was to collect data from a minimum of 40 terminals (10 from each animal), a few additional terminals were analyzed in some groups, explaining why the total number of data points is slightly larger than 20 for some measurements. This issue has been addressed in the revised manuscript (page 10 and 13; the figure number is changed in the revised manuscript: Figure 3, Figure 5, Figure 6 and Figure 7).

13. The legends for Figures 3 and 4 indicate that panels A and B are histograms, whereas they are bar graphs showing individual data points. Only panels C and D for Figure 3 show histograms. Also, the legend repeats part of the description of C-D panels.

We appreciate the reviewer's comments. The figure legend was modified accordingly. Now the Figure number is 5 and 6 in the revised manuscript.

14. The legend for Figure 4 provides the formula for calculating the mitochondrial complexity index, but the superscripts are missing. Please correct.

The formula has been removed from the figure legend. It is now mentioned in the Methods section (page 11; the figure number is changed in the revised manuscript: figure 6) with corrected superscripts.

15. The part of the discussion dedicated to regional differences between the VApc and CM, even in control animals, was somewhat disappointing, given such interesting findings. Clearly much "remains to be established," but is there truly nothing in the literature at present that the observed distinctions might relate to? They are especially interesting given that the same GPi neuron produces both types of axon terminals and that they likely differ as well in their mitochondrial capacity (present study). Is anything known about these parts of thalamus that might indicate local factors affecting the structure and function of extrinsically derived axons? On a related note, are the authors aware of any other reports of mitochondrial differences between collateralized axons known to originate from the same cells? The authors only cite papers describing variance across brain regions.

We thank the reviewer for this important and constructive comment. To our knowledge, very few studies have examined the ultrastructure of axon terminals that originate from single axons throughout the CNS. However, evidence from our laboratory and others showed that axon terminals from single layer V pyramidal neurons that send collateral projections to the striatum and posterior thalamus display strikingly different morphology and pattern of synaptic connections. These findings and other studies related to the development and maturation of nuclei-specific thalamic inputs are discussed on page 18, 19 of the revised manuscript.

16. Please consistently label synapses with arrows in all EM images.

Synapses are now labeled with arrows in all EM images.

17. Add a light microscopic image of the terminals and a drawing depicting the exact position of the EM samples shown in a stereotactic map indicating the AP level.

We thank the reviewer for the comment. As suggested by the reviewer, the light microscopic image of the terminals in VApc and CM and the drawing depicting the exact position of the EM samples along with AP levels have been included in figure 1.

18. The legend of Fig. 2. Reads as follows: "The number of synapses and surface area per Gpi axon terminals are significantly larger in parkinsonian monkeys than in controls (one-way ANOVA with Tukey's post hoc test, ***P = 0.0005; ****P < 0.0001)." A reference to CM is missing from this sentence.

Sorry for the confusion, the modified figure legend includes the CM reference.

19. When the complexity of the pallidal terminals is discussed, a reference to Bodor et al. ,2008 might be considered since this is the first study which reconstructed basal ganglia terminals of primates in 3D. The exact numerical values (no. of synapses, size of terminals correlation between the two) of the two studies can be compared.

We thank the reviewer for this suggestion. As requested, we have added and discussed the results of the Bodor et al., 2008 study in the revised manuscript (page 17).

20. A brief description of mitochondrial complexity index may be appropriate even if a reference is provided to illustrate the changes in mitochondria in representative EM images.

A description of mitochondrial complexity index has been added on page 11 21. RTN-like terminals. A very nice addition to the study is that a group of non-pallidal terminals are shown which does not change their morphology. However, the authors refer to these terminals as GABAergic terminals, but GABA labelling has not been performed. Based on the images provided it is not entirely convincing that purely based on the structure of the postsynaptic density the identity of this group of terminals can be determined. In Fig 5 B,C,D it is not labelled which boutons the authors consider as RTN-like. My suggestion is that either show convincing images about asymmetric and symmetric terminals or use the term "non-pallidal" rather than RTN-like. A brief series of 3-4 EM image may help to clarify this point as well.

As per the reviewer's suggestion, we replaced RTN-like terminals by putative GABAergic non-pallidal terminals throughout the manuscript.

22. Please state in the ms that these reconstructed non-pallidal terminals did not receive afferent inputs thus are unlikely belong to F2 terminals.

As per the reviewer's suggestion we have added a note in the methods section indicating that the non-pallidal terminals are devoid of synaptic inputs, making them unlikely to belong to the F2 terminals group (page 10).

23. The authors employ a synapse-based approach, treating each synapse as an independent data point for statistical analysis. This method artificially inflates the sample size (n) compared to a methodology that would involve averaging data across individual animals. This should be discussed and the authors should clearly state what is their n, and how they have justified it. Also, discussion on the matter is needed.

This is a critical point, and we agree with the reviewer that our approach of treating the number and volume of synapses made by a terminal as an independent data point for statistical analysis has some limitations that are now clearly acknowledged in the revised manuscript (page 11). In this study, we did so because of the limited number of nonhuman primates that could be used, and the large number of observations collected from each animal. To assure that the readers can appreciate the inter-individual differences between data collected from each animal in the same group, the data points collected in each animal are now labeled differently in the graphs. It is noteworthy that the use of the number of elements, instead of the number of animals, in statistical analyses is a common practice for nonhuman primate 3D EM studies (Bodor et al. 2008) and in the field of in vivo and in vitro electrophysiology.

24. There is a lack of functional output on how increase in synapse volume or an increase in mitochondrial complexity index impacts synaptic activity at GPi terminals. Either a mitochondrial activity assay or recordings at these synapses would truly elevate the paper and help understand how these structural changes impact synapse function or more discussion on this matter is needed.

We thank the reviewer for this excellent suggestion. We hope that our findings will lay the foundation and generate interest to launch future functional studies of mitochondria activity and changes in synaptic activity of GPi terminals between the control and parkinsonian states. Because such functional studies are difficult to achieve in nonhuman primates, it will be important to determine if our observations are also found in rodent models of parkinsonism. If so, these could be used to address the important questions raised by the reviewer. We have added relevant information to the discussion about the possible mechanisms through which increases in synapse volume or mitochondrial complexity index may impact synaptic activity of pallidothalamic terminals (Page 21).

25. The assertion regarding structural plasticity in GPi terminals based on target specificity is interesting but needs to further developed to understand the significance of this finding.

As discussed in our reply to point #15, we have now added additional new information to the discussion about the target specificity of morphometric differences between GPi terminals in VApc and CM. Although functional studies are needed to substantiate the impact of our anatomical observations on the properties of pallidothalamic synapses in VApc and CM in normal and parkinsonian state, we believe that the present findings and others now discussed in this paper raise the interesting possibility that individual axons may give rise to morphometrically and functionally diverse populations of target specific axon terminals.

26. visual abstract are missing A visual abstract has been added to this revised submission.
